# The Glutathione Reductase GSR-1 Determines Stress Tolerance and Longevity in *Caenorhabditis elegans*

**DOI:** 10.1371/journal.pone.0060731

**Published:** 2013-04-08

**Authors:** Kai Lüersen, Dirk Stegehake, Jens Daniel, Mike Drescher, Irene Ajonina, Caroline Ajonina, Patrick Hertel, Christian Woltersdorf, Eva Liebau

**Affiliations:** Department of Molecular Physiology, Institute for Animal Physiology, University of Muenster, Muenster, Germany; Virginia Commonwealth University, United States of America

## Abstract

Glutathione (GSH) and GSH-dependent enzymes play a key role in cellular detoxification processes that enable organism to cope with various internal and environmental stressors. However, it is often not clear, which components of the complex GSH-metabolism are required for tolerance towards a certain stressor. To address this question, a small scale RNAi-screen was carried out in *Caenorhabditis elegans* where GSH-related genes were systematically knocked down and worms were subsequently analysed for their survival rate under sub-lethal concentrations of arsenite and the redox cycler juglone. While the knockdown of γ-glutamylcysteine synthetase led to a diminished survival rate under arsenite stress conditions, GSR-1 (glutathione reductase) was shown to be essential for survival under juglone stress conditions. g*sr-1* is the sole GSR encoding gene found in *C. elegans*. Knockdown of GSR-1 hardly affected total glutathione levels nor reduced glutathione/glutathione disulphide (GSH/GSSG) ratio under normal laboratory conditions. Nevertheless, when GSSG recycling was impaired by *gsr-1(RNAi)*, GSH synthesis was induced, but not vice versa. Moreover, the impact of GSSG recycling was potentiated under oxidative stress conditions, explaining the enormous effect *gsr-1(RNAi)* knockdown had on juglone tolerance. Accordingly, overexpression of GSR-1 was capable of increasing stress tolerance. Furthermore, expression levels of SKN-1-regulated GSR-1 also affected life span of *C. elegans*, emphasising the crucial role the GSH redox state plays in both processes.

## Introduction

Aerobic life uses the oxidizing power of O_2_ for various fundamental biochemical processes including energy generation in form of ATP synthesis realised by a controlled stepwise reduction of molecular oxygen. However, oxygen consumption has severe side effects, since, inevitably, reactive oxygen species (ROS) are produced as by-products. If not countered or buffered, these ROS attack and disturb biological molecules and cellular structures leading to a state of impaired physiology also known as oxidative stress. Hence, aerobe organisms have evolved a variety of mechanisms such as antioxidants, detoxification enzymes and repair systems to counteract the deleterious effects of ROS [1], [2]. ROS production and/or intake are further enhanced under pathological conditions with inflammatory implications (e.g. rheumatoid arthritis, diabetes, cancer or neurodegenerative diseases) or by environmental stressors [3], [4]. Moreover, according to the free radical theory of aging, oxidative stress-mediated accumulation of damaged biological molecules favours the aging process and shortens the life span of organisms [5], [6]. In particular, endogenous superoxide radicals derived from mitochondrial respiration have been implicated to be a major cause for aging. In good agreement with that, resistance to oxidative stress was often found to correlate with longevity in different metazoan organisms [7], [8], although some concerns over this linkage have recently been raised [9], [10].

In animal cells, the tripeptide glutathione (γ-glutamylcysteinylglycine, GSH) represents the predominant low molecular weight thiol [11]. Under normal physiological conditions most of the redox-active GSH molecules are reduced and only a minor fraction of the tripeptide is present as glutathione disulphide (GSSG). Accordingly, the GSH/GSSG couple represents a major cellular redox buffer that significantly contributes to the maintenance of the reduced intracellular milieu and, hence, to the anti-oxidative capacity of cells. GSSG, formed when GSH serves as a biological reductant, has to be recycled by the NADPH-dependent GSSG reductase (GSR, EC 1.6.4.2). In addition, intracellular GSH homeostasis is regulated by a synthesis pathway consisting of a two-step reaction catalysed by γ-glutamylcysteine synthetase (GCS) and GSH synthetase (GSS). Furthermore, exogenous GSH was reported to represent an important source to replenish the intracellular GSH pool, however, only after extracellular breakdown and intracellular re-synthesis of the tripeptide via the γ-glutamyl cycle, where the γ-glutamyl transferase (GGT) catalyses the first and rate limiting step. Being a coenzyme or a substrate for diverse enzymes such as glutathione peroxidases (GPX), glutathione S-transferases (GST) and glutaredoxins (GLRX), GSH functions as a central player in redox regulation, ROS defence and phase II detoxification [11], [12].

The nematode *Caenorhabditis elegans* is an established model organism in research on stress defence and aging offering distinct advantages [9], [13], [14], [15]. The worm can be easily cultured on agar plates, reproduces with a rapid life cycle of approximately 3.5 days and has a maximum life span of only about 30 days. *C. elegans* is genetically tractable by RNA interference or germ-line transformation via microinjection allowing the assessment of gene function and related phenotypes at the organismic level. Moreover, in silico analysis of the approximately 19,000 genes revealed that central pathways related to stress defence and aging including putative homologues of the GSH metabolism genes that are well conserved among metazoa are also found in the worm. Consistent with that it has been demonstrated in many studies that the transcription factors *C. elegans* DAF-16 (homologous to the mammalian forkhead box family FOXO) and *C. elegans* SKN-1 (homologous to the mammalian NF-E2 related factor Nrf-2) have a central position in stress resistance and life span determination in metazoan organisms from *C. elegans* up to mammals, being controlled among others by the insulin-like/IGF and p38 MAP-kinase pathways ([14], [16], [17]. DAF-16 and SKN-1 are responsible for the induction of numerous stress response genes including superoxide dismutases, catalases and GSH-related genes such as *gcs* and *gst*.

The highly reactive metalloid arsenite causes oxidative stress by binding directly to thiols such as GSH or by the formation of intracellular ROS [18]. It is well known that the susceptibility of cells towards arsenite correlates with their intracellular GSH level and that treatment with the GCS-inhibitor buthionine sulfoximine diminishes resistance [19]. Biotransformation of inorganic arsenic can either involve repetitive reduction and oxidative methylation, the direct methylation of arsenic-glutathione complexes or direct complexation of trivalent arsenic with GSH. These arsenic-GSH complexes are possibly involved in the efflux of arsenicals form cells by ATP-binding cassette membrane transporters. Futhermore, arsenite and the complexes with arsenite are inhibitors of the GSR, creating a negative feedback loop involving reduction and complexation of trivalent arsenic by GSH and modulation of GSR activity [20], [21].

Black walnut toxicity is due to the allelopathic naphtoquinone Juglone, that is being used in natural dyeing, as herbicide, dietary supplement and in various folk medicines to treat bacterial, fungal or viral infections of the skin. Its toxicity, and probably also its antibiotic, antiviral, and antifungal properties are due to the intracellular ROS production. Juglone is able to cross cell membranes and generate superoxide anion from molecular oxygen during metabolism [22], [23], [24]. Juglone response in *C. elegans* has frequently been analysed by investigating alterations in transcript levels and reporter gene expression. Here a number of genes/pathways that are affected under juglone exposure were revealed, including genes of the GSH metabolism [23], [25], [26], [27], [28], [29]. However, the induced or decreased expression of a particular gene or even a protein is not the ultimate evidence of its essential role in stress tolerance of an organism.

In the present study we have employed a small scale RNAi screen in *C. elegans* to address the question which components of the GSH-metabolism are required for the tolerance towards sub-lethal intracellular oxidative stress generated by arsenite and the redox cycler juglone. Here, it is important to note that knockdown by RNAi can be variable and can lead to false negative results. Since only the induction of a detectable phenotype is a reliable indicator of a positive RNAi result, only these enzymes will be considered. Furthermore, our experimental setup cannot exclude combined effects of several enzymes.

Whereas tolerance towards arsenite was shown to be GSH synthesis-dependent, the gene C46F11.2 encoding GSR-1, a protein orthologous to human mitochondrial glutathione reductase, was identified to be absolutely essential for survival under sub-lethal juglone stress. Our studies revealed that expression levels of SKN-1-regulated GSR-1 determine not only the stress tolerance (primarily against endogenous oxidative stress) but also the life span of *C. elegans*, emphasising the crucial role the GSH redox state plays in both processes.

## Materials and Methods

### Oligonucleotides

Oligonucleotide sequences are listed in the **Table 1**.

**Table 1:** Oligonucleotide sequences.

Primer name	Sequence 5′ to 3′	Restriction sites
Primer for GSR cloning:		
CeGR-EX-S	CATATG TCTGGCGTCAAGGAGTTCG	NdeΙ
CeGR-EX-AS	CTCGAG TTATTCCGGCTCACACCTCC	XhoΙ
CeGR-5165-S	CTGCAG CCATCATTTCGGGACTCGACATTCCGC	PstI
CeGR-5166-AS	GGATCC CAAATAGTCGAACTCCTTGACGCCAGCCAGACATGATCGA	BamHI
CeGR-5306-S	ATGCTCCGATTTCGCTGCATTTTGAGCAC	–
CeGR-5307-AS	TTATTCCGGCTTCACACCTCCTCGCA	–
CeGR-5326-AS	CCCGGG CCTTCCGGCTTCACACCTCC	SmaI
Primer for RNAi clones:		
GST-11-S	CTGCAG AGCCCGCAAGGCTTATCTTC	PstI
GST-11-AS	CTCGAG TTGGGCGAGAGTTGATCCAC	XhoI
GST-17-S	CTGCAG GCGAGGCTCATGTTCCATTC	PstI
GST-17-AS	CTCGAG TTGGCTGCTCTTCAGCCTTG	XhoI
GST-35-S	CTGCAG TGGTGGAGTTCCATTTGAAG	PstI
GST-35-AS	CTCGAG TCAACTCTGGAAGTCCATAG	XhoI
GST-43-S	CTGCAG TCATATTGGCGTTCGTCGTG	PstI
GST-43-AS	CTCGAG TGGCTGATTATCTGGATGAG	XhoI
GSTO-1-S	CCCAAGCTTATGGTTTTAACCGGAGTAAC	HindIII
GSTO-1-AS	CCGCTCGAGTGACGGCGAAGAGCAATGGAA	XhoI
GLRX-3-S	TGGCGGCCGCTCTAGACCAATTCAAGAAATCAAGTCAGG	XbaI
GLRX-3-AS	GCGTCACGTGGCTAGCATCATTTCTAATTCTTGGAATCT	NheI
GLRX-22-S	GCGCTCTAGAGGAGGATCTGCATCAACACCA	XbaI
GLRX-22-AS	GCGCGCTAGCAATTTTCGATGCATATTCTCTCCG	NheI

Underlined nucleotides correspond to restriction sites.

### *C. elegans* Culturing and Strains

*C. elegans* were cultured at 20°C under standard conditions on nematode growth medium (NGM) seeded with Escherichia coli OP50 (Caenorhabditis Genetics Center) as a food source [30] unless otherwise noted. Worm populations were synchronized by alkaline hypochlorite lysis (40% sodium hypochlorite, 5% 10 N NaOH) [31]. The following strains were obtained from the Caenorhabditis Genetics Center at the University of Minnesota, which is funded by the NIH National Center for Research Resources: Wild-type N2 Bristol, GE23 pha-1(e2123)III, TJ356 zIs356 IV [DAF-16::GFP], CL2166 dvIs19[pAF15(*gst-4*::GFP::NLS)III], zcls14[*myo-3*::GFP(mit)]. The strains N2 Ex003[*gcs-1*::GFP] and N2 Is007[SKN-1::GFP] [32], [33] were kindly provided by Keith Blackwell (Harvard Medical School, Boston). The balanced mutant *grs-1* strain VB2729 (*gsr-*1(tm3574)/hT2[*bli-4*(e937) *let*(q782) qIs48]) was kindly provided by Simon Tuck (Umeå Center for Molecular Medicine, Umeå University).

### RNAi Assays

RNAi was induced by feeding worms with *E. coli* HT115 strains producing dsRNA following standard protocols [34]. The bacterial RNAi clones were grown in Luria-Bertani (LB) medium containing 50 µg ml^−1^ ampicillin and 12.5 µg ml^−1^ tetracyclin for 16 h at 37°C, before being spread on fresh NGM agar plates supplemented with 50 µg ml^−1^ ampicillin and 2.5 mM isopropyl *β*-D-thiogalactoside (IPTG). Worms were transferred to RNAi plates either at L4 stage and F_1_ L4/young adults were analysed or as eggs/L1 and the adult worms of the same generation were inspected. Greatest care was applied to avoid contaminating bacteria and/or fungi during feeding of worms. Furthermore, to assess the efficiency of the feeding RNAi method, we used *dpy-5* and *bli-1* as target genes. RNAi resulted in either a dumpy or blistered phenotype.

### Identification of GSH Metabolism Genes and RNAi Screen for Juglone Tolerance

Genes that are potentially involved in the GSH-metabolism of *C. elegans* were identified by combining database mining of annotated genes and Blast searches of WormBase using known genes of GSH-metabolism from other organisms. In total, 67 genes were identified that encode proteins of GSH-synthesis (*gcs-1*, *gs-1*) and GSH-redox cycle (*gpx-1* to *gpx-6*, *gsr-1*), GST (*gst-1* to *gst-44*), GGT (*ggt-1* to *ggt-6*) and GLRX (*glrx-5*, *glrx-10*, *glrx-21*). Most of the *E. coli* HT115 strains that produce the corresponding dsRNA were found in the RNAi library (Geneservice, Cambridge, UK). For genes that were not present in the library, open reading frames were amplified from *C. elegans* cDNA by polymerase chain reaction (PCR) using the oligonucleotides listed in **Table 1**. PCR products were cloned into pL4440 using standard methods. Positive clones validated by DNA-sequencing, were transformed into *E. coli* HT115 cells. Stress tolerance assays with RNAi treated animals were performed as follows: In pre-tests we determined a 90% survival rate of wildtype worms at a juglone concentration of 0.15 mM for an incubation time of 18 h. Using a NaAsO_2_ concentration of 3 mM ensured 80% survival of wildtype worms after 18 h incubation. RNAi assays were carried out as described in [25]. F_1_ young adults were transferred to RNAi-plates supplemented with 0.15 mM juglone. Animals were cultured for additional 18 h at 20°C. The percentage of survivors was determined.

### Nucleic Acid Preparation and Northern Blot Analysis

Synchronised *C. elegans* N2 wild-type worms were cultured on NGM agar under standard conditions until they reached the L4/young adult stage. Animals were allocated and cultured on standard control plates or in the presence of 10 µM juglone for 3 h before being harvested. Total RNA was prepared using TRIzol extraction according to the manufacturer’s instructions (Invitrogen, Carlsbad, CA, USA). For Northern blotting, total RNA was separated on an agarose formaldehyde gel and transferred to a positively charged nylon membrane (Millipore Co., Bedford, MA, USA). The membrane was hybridized with a radio-labelled probe of the *gsr-1* coding region (amplified from *C. elegans* cDNA by using the primer pair *Ce*GR-5306-S und *Ce*GR-5307-AS, **Table 1**) in 50% formamide, 5x standard saline citrate (SSC), 5x Denhardt’s solution, and yeast tRNA at 55°C overnight, followed by washing with 2x SSC and 0.1% sodium dodecyl sulfate (SDS) at 60°C.

### Plasmid Constructs and Transgenic *C. elegans*

#### Pgsr-1::GFP

A 2.4 kb fragment including 1996 bp of the 5′ upstream region, the first exon, the first intron and part of exon II of *gsr-1* was amplified from the *C. elegans* genomic DNA using Expand Long Template PCR system (Roche, Mannheim, Germany) and the primers *Ce*GR-5165-S and *Ce*GR-5166-AS5 (**Table 1**). The PCR product was cloned into pPD95.77 provided by A. Fire (Carnegie Institute, Baltimore, USA).

#### Pgsr-1::gsr-1::GFP

A 3864 kb genomic fragment that includes 1996 bp of the 5′ upstream region and the complete open reading frame of *C. elegans gsr-1* was amplified by PCR using the primers *Ce*GR-5165-S and *Ce*GR-5326-AS (**Table 1**). The PCR product was cloned into pPD95.77.

Germline transformation with the constructs *Pgsr-1::GFP* and *Pgsr-1::gsr-1::GFP* were performed by co-injecting the vector constructs (20–50 µg ml^–1^) with the pRF4 plasmid (80 µg ml^–1^) encoding the dominant marker gene *rol-6* into the germline of young N2 wild-type adults. The *Pgsr-1::gsr-1::GFP* vector construct (80 µg ml^-1^) was introduced into *C. elegans pha-1(e2123)* mutants together with the pBX plasmid (80 µg ml^−1^) containing the dominant marker gene *pha-1* [35]. The *pha-1*/pBX system (a kind gift from R. Schnabel, Braunschweig, Germany) allows thermo-selection of transgenic animals. Only transformed progeny survive and can be easily maintained by cultivation at 25°C. To investigate the cell-specific, developmentally regulated transcription of *gsr-1*, GFP expression patterns were analysed by fluorescence microscopy.

### Analyses of gsr-1 Promoter Activity under Stress Conditions

To analyse alterations in *gsr-1* promoter activity, worms carrying the *Pgsr-1::GFP* reporter construct were allowed to grow from L1 to the L4/young adult stage in the presence of *skn-1(RNAi)*, *daf-16(RNAi)* or control RNAi bacteria before being transferred to corresponding (i) standard RNAi plates, (ii) RNAi plates supplemented with 150 µM juglone or (iii) starvation plates without bacteria. After 16 h incubation worms were inspected for GFP expression intensities.

### GFP Reporter Evaluation

Expression of the intestinal promoter reporters *Pgcs-1::GFP* and *Pgst-4::GFP* as well as of the intestinal nuclear translocation reporters *DAF-16::GFP* and *SKN-1::GFP* was analysed and scored following the method described in Tullet *et al*. [16]. P values were determined from a Fisher test.

### Stress Tolerance Assays

For stress tolerance assays synchronised N2 wildtype L1 larvae were fed with *gsr-1(RNAi), gcs-1(RNAi)* or pL4440 control bacteria under standard RNAi conditions until they reached L4. Worms were then transferred to RNAi plates supplemented with 3 mM cumene hydroperoxide, 0.15 mM juglone, 50 mM paraquat, 7.5 mM tert-butylhydroperoxide or 3 mM NaAsO_2_. Similarly, L4 worms overexpressing GSR-1 in the *pha-1(e2123)* background and promoter::GFP control worms were transferred to NGM plates containing 0.25 mM juglone. Animals were cultured for another 16 h, before their survival rate was evaluated. A worm was scored as dead when it did not respond to a mechanical stimulus. Each experiment was performed at least three times, and the data were analysed using GraphPadPrism software.

### Determination of Life Span and Brood Size

Phenotype analyses of *gsr-1(RNAi)* and *pL4440(RNAi)* control worms were performed at 20°C. Transgenic worms that carry the thermo-selectable pBX in the *pha-1(e2123)* genetic background were maintained at 25°C. To determine brood sizes, worms were cultured individually on NGM plates. Adults were transferred daily to new plates until egg production ceased. Plates with progeny were retained and counted when larvae reached L3/L4. For life span assays, synchronised worms were incubated at 20°C until they reached adulthood. 100 fluorescent (*Pgsr-1::gsr-1::*GFP or *Pufc-1*::GFP) worms were transferred on 10 NGM plates and cultured at 20°C. For the lifspan assays under RNAi-mediated knockdown, synchronized eggs were transferred on RNAi-NGM plates (5 plates with 10 eggs each). The assays were performed independently at least three times. Worms were examined daily and were scored as dead, when they no longer responded to touch-provoked movement. Animals that crawled away from plates or died from internal hatching of progeny were replaced by worms from a parallel substitutes’ plate. Survival plots were compared using the log-rank test (GraphPadPrism software).

### Cloning, Expression, Purification and Enzymatic Activity Assay of the Recombinant GSR-1

The coding region of GSR-1 was amplified from *C. elegans* cDNA by PCR using the oligonucleotides *Ce*GR-EXS and *Ce*GR-EXAS **(** **Table 1**). The PCR product was cloned into the prokaryotic expression plasmid pJC40 [36] and transformed into the *E. coli* strain BL21(DE3) (Novagen, Madison, WI, USA). After expression, recombinant GSR-1 was purified from the supernatant by chelating chromatography on Ni^2+^-nitrilotriacetate (Ni-NTA) agarose according to the manufacturer’s instructions (Qiagen, Hilden, Germany). Subsequently, GSR-1 was subjected to fast protein liquid chromatography on a Superdex 75 column (Amersham-Pharmacia Biotech, Piscataway, NJ, USA). The protein concentration was determined by the method of Bradford [37] and the homogeneity of the enzyme preparation was analysed with 12.5% SDS-PAGE. Proteins were revealed by Coomassie Blue staining or Western blot analysis.

The activity of the GSR-1 was determined spectrophotometrically at 25°C (UVIKON-932 spectrophotometer, Goebel, Germany) as previously described by Müller *et al.* [38]. The decrease of absorbance at 340 nm due to the oxidation of NADPH was recorded. Background rates were determined in the absence of GSSG. The activity that catalyzes the consumption of 1 µmol of substrate per minute was defined as 1 U. To determine the steady-state kinetic parameters, the enzyme assay was carried by varying the concentration of one substrate (10–500 µM for NADPH and 10–200 µM for GSSG) in the presence of saturating concentration of the other.

### Glutathione Determination

Synchronised three-days-old young adult worms were cultured under RNAi conditions on plates containing (i) control HT115 carrying empty pL4440 vector and (ii) plus 150 µM juglone or (iii) *gsr-1(RNAi)* feeding bacteria and (iv) plus 150 µM juglone. After 4 h incubation, worms were harvested and the GSH redox states and γ-glutamylcysteine level were determined by high-performance liquid chromatography (Spectrasystem P2000 pumps, Thermo Fisher) and fluorometric detection (Jasco 821-FP, Jasco) according to Neuschwander-Tetri & Roll [39]. Briefly, worms were homogenized with a glass-glass Potter-Elvehjem tissue grinder in 100 mM Tris-HCl, pH 8.5 containing 10 mM DTT. For the determination of GSSG, worms were homogenized in the presence of 2 mM N-ethylmaleimide to eliminate free thiol-groups, before the addition of DTT. Proteins were precipitated by 2 N perchloric acid. Thiols were derivatized for two minutes with orthophthalaldehyde (OPA-reagent) in 0.4 M potassium borate, pH 9.9 before samples were neutralized by the addition of 100 mM sodium phosphate, pH 7.0. Separation of OPA-labeled thiols were performed isocratically on a Nucleosil 120-5 C18 column (250×3 mm; Macherey-Nagel) at a flow rate of 0.55 ml min^−1^ with 7.5% methanol, 92.5% 150 mM sodium acetate buffer, pH 7.0. Peaks were detected at 420 nm after excitation at 340 nm.

## Results

### GSR-1 is Required for Tolerance Towards the Pro-oxidant Juglone

A survey based on homology searches and already confirmed gene functions revealed 67 genes that are directly involved in the GSH metabolism of *C. elegans* (**Table 2**). Microarray analyses have demonstrated that the transcription level of numerous of these genes are altered under ROS and other stress conditions [40]. However, changes in mRNA levels do not indicate whether a gene is required for organismic tolerance towards a stressor. To address this question, we carried out a small scale RNAi screen where *C. elegans* was exposed to sub-lethal concentrations of the pro-oxidant juglone. Among the 67 GSH-metabolism genes tested only C46F11.2 was found to be absolutely essential for juglone tolerance (juglone survival rate 3.3±7.3, *p*<0.001, **Table 2**); a more moderate effect was observed with the previously characterised omega-class *gst*, GSTO1 (juglone survival rate 16.4±1.7, *p<*0.01) [25].

**Table 2:** Small scale RNAi screen testing juglone and arsenite tolerance.

Gene	Annotation	RNAi clone	Juglone survival	Arsenite survival
Control	–	L4440	90.8±7.4	90.0±5.8
*γ-Glutamylcysteine synthetase (γ-GCS)*				
F37B12.2	*gcs-1*	II-6D11	67.8±1.9	0***
*Glutathione synthetase (GSS)*				
M176.2	*gss-1*	II - 6P23	85.6±13.5	76.7±14.1
*Glutathione reductase (GSR)*				
C46F11.2^+^	*gsr-1*	III-1H04	3.3±7.3***	87.8±8.4
*Glutathione S-transferases (GST)*				
R107.7	*gst-1*	III-4J12	94.7±5.1	76.7±4.7
K08F4.6	*gst-2*	IV-5I02	93.3±2.7	74.5±7.8
K08F4.11	*gst-3*	IV-5I10	91.7±1.9	81.5±2.1
K08F4.7	*gst-4*	IV-5I04	94.2±5.7	78.3±11.8
R03D7.6^+^	*gst-5*	II-7H21	94.2±5.7	63.3±26.5
F11G11.2	*gst-7*	II-4E11	82.5±11.0	76.7±14.5
F11G11.1	*gst-8*	II-4E09	88.3±8.4	58.9±15.8
R05F9.5	*gst-9*	II-4I01	83.3±7.2	64.4±16.8
Y45G12C.2	*gst-10*	V-2E10	89.2±12.6	93.3±9.4
R11G1.3	*gst-11*	cloned	88.1±10.3	n.d.
F37B1.2	*gst-12*	II-8F10	85.0±10.3	56.2±22.9
T26C5.1	*gst-13*	II-6J13	89.2±9.6	91.7±7.1
F37B1.3	*gst-14*	II-8F12	83.3±8.2	67.8±10.2
F37B1.4	*gst-15*	II-8F14	81.7±10.0	90.0±1.5
F37B1.5	*gst-16*	II-8F16	90.0±7.2	78.7±7.1
F37B1.6	*gst-17*	cloned	96.4±3.6	n.d.
F37B1.7	*gst-18*	II-8F18	89.2±9.8	75.0±7.1
F37B1.8	*gst-19*	II-8F20	85.8±13.2	78.3±21.2
ZK697.6^+^	*gst-21*	V-1J14	77.5±14.8	66.7±17.6
F21H7.1	*gst-22*	V-10H12	83.3±12.5	85.0±11.8
T28A11.11	*gst-23*	V-2N04	92.5±6.9	83.3±4.7
F37F2.3	*gst-25*	I-1O23	86.7±4.7	96.7±0.0
Y53F4B.29	*gst-26*	II-9O02	91.1±6.9	73.3±23.6
Y53F4B.30	*gst-27*	II-9O04	87.8±1.9	90.0±9.4
Y53F4B.31	*gst-28*	II-9O06	92.2±7.7	91.7±2.4
Y53F4B.32	*gst-29*	II-9O08	91.1±5.1	47.0±26.7
Y53F4B.35	*gst-31*	II-9O14	91.1±6.9	71.7±16.5
Y53F4B.37	*gst-32*	II-9O18	87.8±6.9	24.0±13.2**
Y1H11.1	*gst-34*	V-10H12	85.6±8.4	63.3±2.3
Y1H11.2	*gst-35*	cloned	94.6±2.1	n.d.
R07B1.4	*gst-36*	X-4J08	87.8±5.1	77.3±10.4
Y32G9A.1	*gst-37*	V-13E20	92.2±3.8	73.3±1.0
F35E8.8	*gst-38*	V-10F05	91.1±6.9	40.0±17.6
Y53F4B.33	*gst-39*	II-9O10	91.9±8.8	88.3±7.1
F56B3.10	*gst-40*	IV-1E20	88.6±11.7	63.3±23.6
R13D7.7	*gst-41*	V-5I06	89.8±3.7	78.3±2.4
D1053.1	*gst-42*	X-5J07	91.1±9.6	60.8±12.4
Y71F9AL.5	*gst-43*	cloned	95.5±1.1	n.d.
F13A7.10	*gst-44*	V-10P14	90.0±3.3	15.0±6.4***
C29E4.7	*gsto-1*	cloned	16.4±1.7**	86.5±9.0
C02D5.3	*gsto-2*	III-4F21	80.0±3.3	64.1±27.4
C25H3.7	*gst-like*	II-4H13	90.0±8.8	78.3±11.8
R11A8.5	*gst-like*	IV-5B15	81.1±9.6	85.3±3.5
*γ-Glutamyltransferase (γ-GT)*				
T03D8.6^+^	*-*	V-13A06	83.3±23.1	70.0±4.7
C53D5.5	-	I-1C15	90.0±5.8	70.0±4.7
Y7A9A.1	-	IV-7H18	84.4±5.1	46.7±23.3
H14N18.4^+^	-	V-6E10	77.8±24.1	58.9±22.2
Y97E10AR.2^+^	-	V-13B05	92.2±7.7	75.0±11.8
T19H12.6	-	V-3L18	86.7±8.8	58.9±17.1
*Glutathione peroxidase (GPX)*				
F26E4.12	*gpx-1*	I - 4L14	96.7±3.3	60.0±18.9
C11E4.2	*gpx-3*	X - 4D12	90.0±11.5	83.3±4.7
C11E4.1	*gpx-5*	X - 4D10	80.0±14.5	13.3±9.4***
T09A12.2^+^	*gpx-6*	IV - 4M01	84.4±11.7	71.7±2.4
R03G5.5^+^	*gpx-7*	X - 4E07	91.1±3.8	61.7±21.2
F55A3.5	*gpx-8*	I - 5O10	92.2±3.8	66.7±23.6
*Glutaredoxin (GLRX)*				
D2063.3^+^	*glrx-3*	cloned	89.3±0.5	96.6±1.0
Y49E10.2	*glrx-5*	III - 6C14	88.9±10.7	53.7±27.8
Y34D9A.6	*glrx-10*	I - 7D15	90.0±8.8	83.3±14.1
ZK121.1^+^	*glrx-21*	III - 2F14	94.4±3.8	79.8±9.2
C07G1.8	*glrx-22*	cloned	94.8±9.8	81.8±2.1
*Phytochelatin synthase (PCS)*				
F54D5.1^+^	*pcs-1*	II - 7J22	78.1±8.5	89.1±1.4
*Glyoxalase (GLOD)*				
C16C10.10	*glod-4*	III - 2G01	87.9±11.2	82.7±2.0

Genes involved in the *C. elegans* GSH-metabolism were classified according to their reported or predicted functions. Each of the 67 listed genes was targeted by RNAi starting in L4 animals. F_1_ L4/young adults of the treated worms were transferred to 0.15 mM juglone and 3 mM arsenite stress plates, respectively, and the survival rates (± SD) were determined after 18 h at 20°C. Significance levels were determined by student’s t-test (n ≥3, at least 90 animals, *P<0.05, **P<0.01, and ***P<0.001). RNAi clones of the RNAi library (Geneservices, positions are shown) were verified by PCR using T7-primer followed by analytical restriction. Where indicated, RNAi constructs for genes that are not included in the library were generated by standard cloning of PCR products into the pL4440 vector, n.d. not determined.^+^genes possess different isoforms, however, all isoforms are affected by the RNAi constructs used.

C46F11.2 encodes a member of the pyridine nucleotide-disulphide oxidoreductase family orthologous to human mitochondrial GSR (www.wormbase.org) and shares 65% and 58% amino acid sequence identity with the GSR from the filarial nematode *O. volvulus* and humans (data not shown). g*sr-1* is the sole GSR encoding gene found in *C. elegans*. The gene contains 5 distinct gt-ag introns and transcription produces four alternatively spliced mRNAs (C46F11.2a, C46F11.2b1-3). Whereas the long transcript C46F11.2a, starting from exon 1 has a coding region of 1422 bp (473 aa), the shorter transcripts C46F11.2b1-3, encompassing exons 2–5, have a coding region of 1380 bp (459 aa). As with other anti-oxidant factors, the *gsr-1* is clearly expected to reside in the cytosol and mitochondria. Since both a mitochondrial and a cytosolic isoform of the GSR-1 could be generated from a single gene through alternative splicing, analysis of the long isoform was performed, using PSORT [41]. This program allows the prediction of protein localization sites and a mitochondrial localization with a probability of 43.5% was calculated. This is in agreement with the MITOPROT analysis [42], however, a mitochondrial matrix peptidase cleavage site could not be predicted. In worms carrying the *Pgsr-1::gsr-1::GFP* reporter construct (1996 bp of the 5′ upstream region and the complete open reading frame of *gsr-1* tagged with GFP), only strong cytosolic GFP expression was observed, possibly obscuring lesser granular mitochondrial GFP signals.

Since C46F11.2 had not been characterised before, we cloned the corresponding open reading frame of 1380 bp for recombinant expression in *E. coli*. Consistent with the theoretical molecular mass, the purified His-tag fusion protein that forms an enzymatically active homodimer had a molecular mass of approximately 52 kDa (**Figure S1 A**). The protein catalysed the reduction of GSSG by using NADPH as an electron donor. The K_m_ values for GSSG and NADPH were found to be 34.1 µM and 12.9 µM, respectively (**Figure S1 B**) indicating that C46F11.2 encodes a functional GSR.

Initially we wanted to use the balanced gsr-1 knockout strain (allele tm3574, mutation site: 9752/9753-10135/10136), however, even under normal laboratory conditions, the impairment of homozygous sterile mutants was so severe, making stress experiments futile. Therefore, to further evaluate the role of *C. elegans* GSR-1 in stress tolerance, *gsr-1(RNAi)* worms were exposed to environmental stressors. To demonstrate the effect of *gsr-1(RNAi)* on *gsr-1* expression levels, *gsr-1(RNAi)* was performed on *Pgsr-1::gsr-1::GFP* worms. Here, the dimmed GFP fluorescence signal and a reduced amount of GSR-1::GFP clearly indicates the efficiently reduced *gsr-1* expression (**Figure S2).**

**Figure 1:**
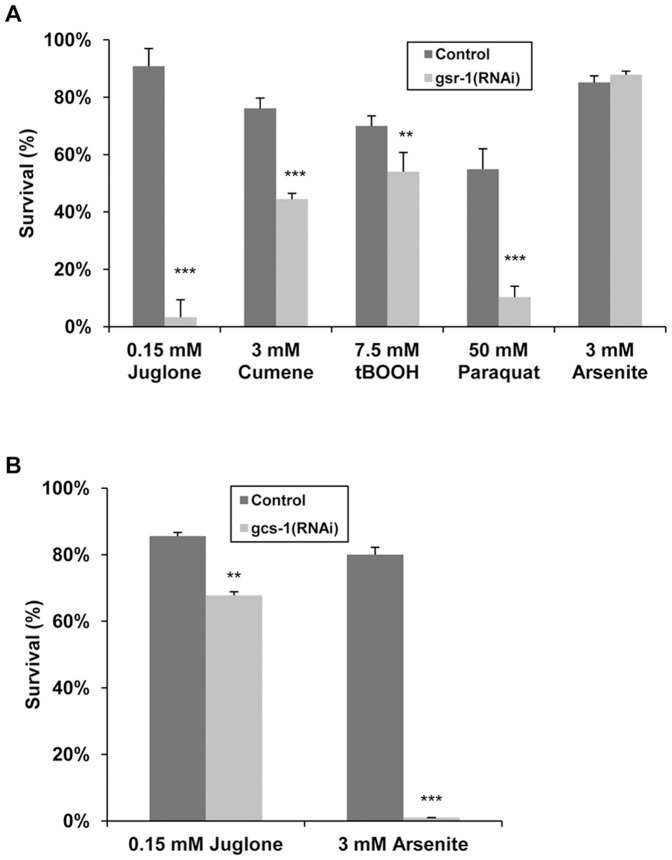
Analyses of GSR-1 and GCS-1 dependent stress tolerance. Wild-type eggs/L1 were placed on RNAi plates (A) *gsr-1(RNAi)* or (B) *gcs-1(RNAi)* and cultured until they have reached the L4/young adult stage, before being transferred to corresponding RNAi plates containing the indicated stressors. As control, plates with empty control vector pL4440 were used. Following 16 h incubation at 20°C, the survival rate was determined. Data represent means of at least three independent triplicate determinations (n≥120 animals, p values from Fisher test).

As shown in **Figure 1A**, GSR-1 was also found to be crucial for the tolerance against other pro-oxidants including paraquat and to a lesser extent cumene hydroperoxide and tert-butylhydroperoxide. Knockdown of GSR-1, however, did not affect the sensitivity of *C. elegans* towards arsenite. Remarkably, protection against this stressor required the presence of the GSH synthesis enzyme *gcs-1* (**Figure 1B**). In contrast to that, RNAi-mediated inhibition of GCS-1 hardly altered the susceptibility to juglone.

### GCS and - to a Lesser Extend gst-32, gst-44 and an Extracellular GPX C11E4.11 - are Required for Tolerance Towards Arsenite

Several studies have shown that GSH synthesis plays a critical role in the protection of *C. elegans* against inorganic arsenite, with GCS-1 being involved in the protection [43], [44]. We can confirm that the first rate-limiting enzyme of GSH synthesis, the GCS is essential for protection (0% survival rate of *gcs(RNAi)-*worms under the given arsenite concentration) (**Figure 1b**). Furthermore, knockdown of an alpha-class related GST (*gst-32*), an omega-class GST (*gst-44*) and an extracellular glutathione peroxidase (C11E4.1) less profoundly affected the survival rate under arsenite stress (arsenite survival rate of 24.0±13.2 (*p<*0.01), 15.0±6.14 (*p<*0.001) and 13.3±9.4 (*p<*0.001), respectively). Knockdown of *gsr-1* expression had no effect on the survival rate of worms under arsenite stress conditions (**Table 2**). However, this negative result must be treated with caution since small amounts of residual GSR-1 might be sufficient for arsenite tolerance.

### The Effect of gsr-1(RNAi) and Juglone Stress on the GSH-redox State

**Figure 2:**
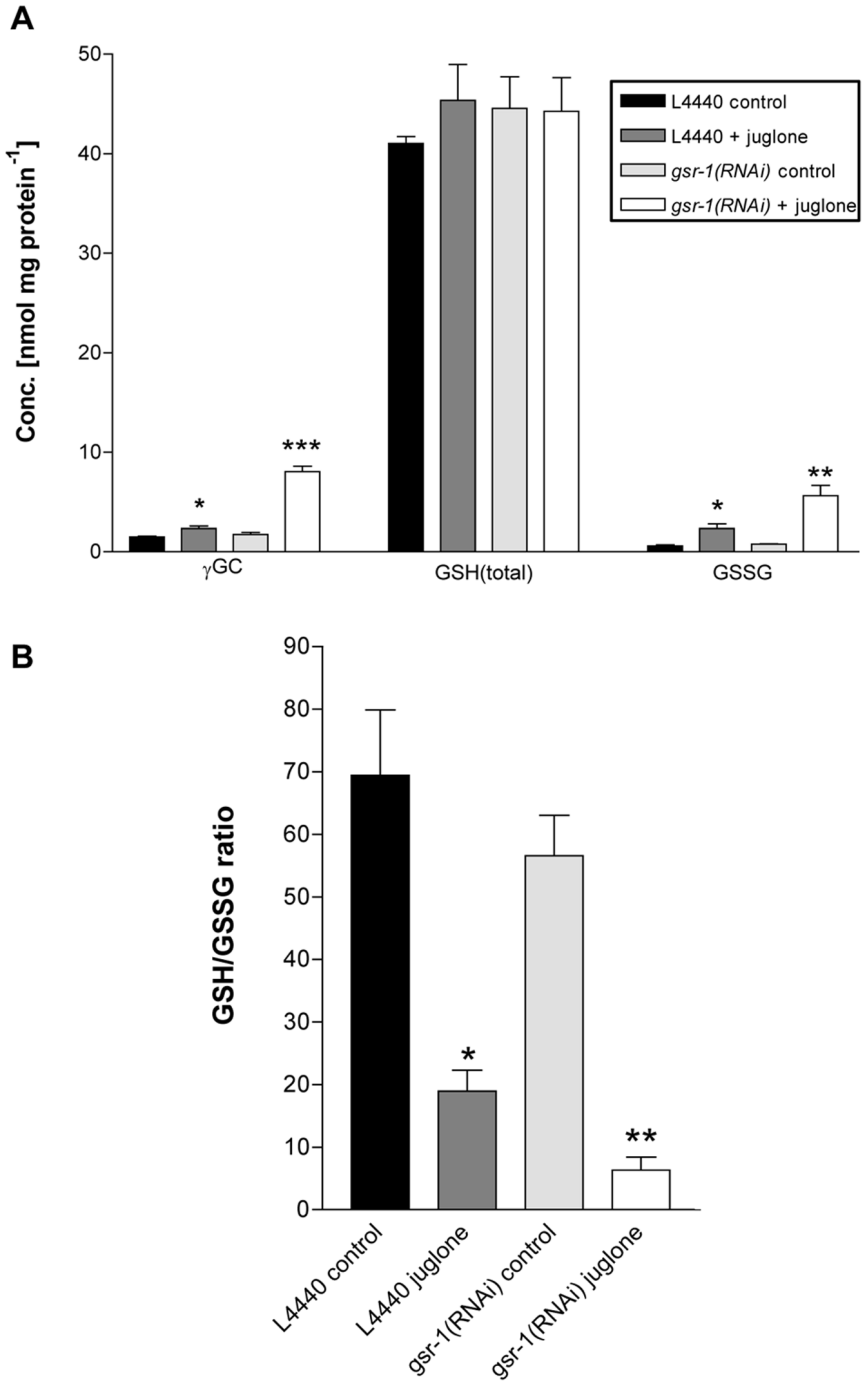
Determination of γ-glutamylcysteine, GSH and GSSG levels in lysate of synchronised 3-days-old worms. (**A**) Worms were cultured on RNAi-plates containing (i) HT115 bacteria carrying empty pL4440 vector or (ii) HT115 bacteria carrying empty pL4440 vector plus 150 µM juglone or (iii) *gsr-1(RNAi)* feeding bacteria or (iv) *gsr-1(RNAi)* feeding bacteria plus 150 µM juglone. (**B**) Analysis of the respective GSH/GSSG ratios.

The physiological role of GSR is the recycling of oxidized GSSG to GSH, thereby maintaining the intracellular GSH redox state, which is essential for cellular integrity. To test the role of *C. elegans gsr-1* in the regulation of the redox state, we therefore analysed the GSH/GSSG ratio in *gsr-1(RNAi)* worms (**Figure 2**). Interestingly, knockdown of GSR-1 hardly affected the total GSH-level of about 40 nmol/mg protein, nor the GSH/GSSG ratio found in control worms (70∶1). However, when worms were exposed to oxidative stress using juglone, the effect on GSH-related metabolites, that is enhanced GSSG- and γ-glutamyl-cysteine (γ-GC) levels and reduced GSH/GSSG ratios, was potentiated (**Figure 2**). The GSH-precursor γ-GC changed from 2.3 to 8.1 nmol/mg protein, indicating that GSH-synthesis is boosted under oxidative stress conditions. Simultaneously, the GSSG-concentration altered from 2.0 to about 6.0 nmol/mg protein in control worms and *gsr-1(RNAi)* worms exposed to juglone stress, leading to GSH/GSSG ratios of about 20∶1 and 6∶1, respectively. This explains the enormous effect of *gsr-1* knockdown on the tolerance towards juglone stress observed in the above mentioned results from the juglone toxicity RNAi-screening (**Table 2**).

### Expression Pattern of Stress-inducible GSR-1

**Figure 3:**
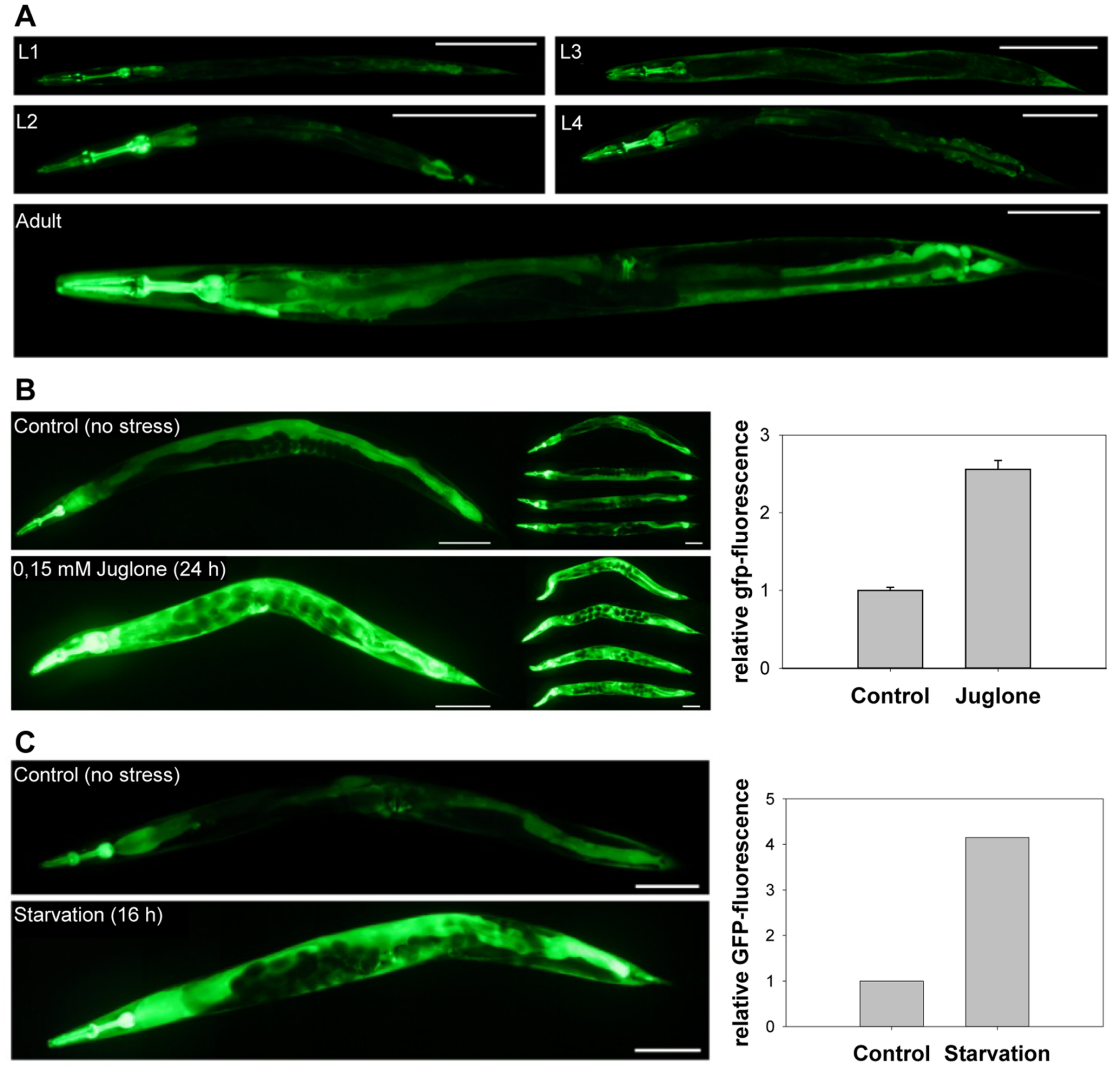
GSR-1 expression pattern under standard and stress conditions. (**A**) The GSR-1 expression pattern of all stages was examined under standard culture conditions using *the Pgsr-1::GFP* worms. L4/young adults were transferred to (**B**) NGM plates containing 0.15 mM juglone. (**C**) Effect of dietary deprivation on *gsr-1* promoter activity. *Pgsr-1::GFP* worms were grown under standard culture conditions until they reach the L4/young adult stage. Animals were then transferred to NGM agar plates without *E. coli* food for 16 h, before being analysed by fluorescence microscopy (quantified by ImageJ). Note the different scale bars for L1–L3 (50 µm), for L4 and adult (100 µm).

To analyse the temporal and spatial expression pattern of *C. elegans gsr-1*, we generated transgenic *Pgsr-1::GFP* and *Pgsr-1::gsr-1::GFP* worms expressing the GFP reporter under the control of the *gsr-1* promoter. In both cases bright fluorescence was seen mainly in the intestine, vulval muscle, the pharynx and some unidentified cells in the tail region (**Figure 3A**). Although the overall spatial GFP expression pattern was similar throughout all postembryonic life stages, a general increase in the fluorescence signals was observed during the development from L1 to adult worms. As control, *gsr-1(RNAi)* on *Pgsr-1::gsr-1::GFP* worms was performed and efficiently reduced *gsr-1* expression as indicate by a dimmed GFP fluorescence signal and a reduced amount of GSR-1::GFP fusion protein (**Figure S2)**.

**Figure 4:**
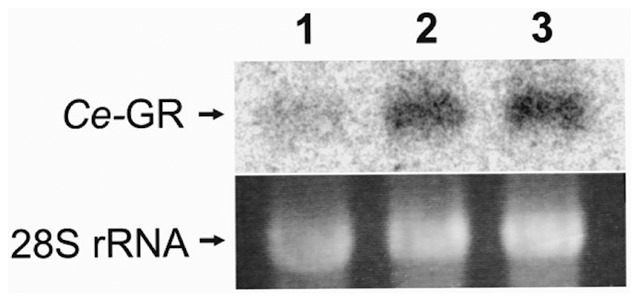
Northern blot analyses. *C. elegans* N2 wild-type worms were cultured on NGM agar under standard conditions (lane 1) or exposed to 150 µM (lane 2) and 300 µM juglone for 4 h, before total RNA was isolated and applied to Northern blot analyses using a radiolabeled *gsr-1* probe. Since there is only a 40 bp difference in transcript size (C46F11.2a, C46F11.2b1-3), only one band can be observed.

Notably, the high GFP expression level was further elevated when the reporter strains were exposed to juglone (**Figure 3B**). Northern blot analyses of N2 wildtype worms confirmed the enhanced *gsr-1* expression in the presence of the naphthoquinone stressor (**Figure 4**).

### Juglone and Starvation Induced gsr-1 Expression is Regulated by SKN-1, but not by DAF-16

**Figure 5:**
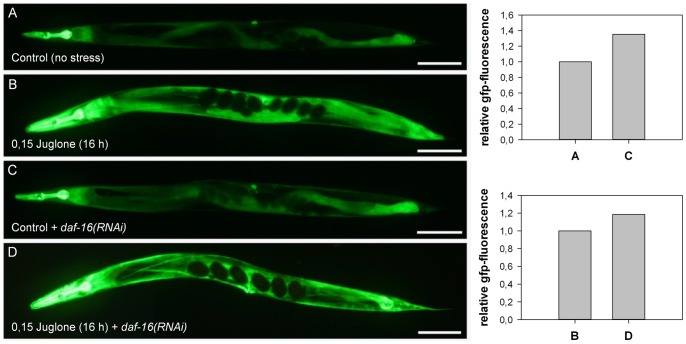
Juglone-induced GSR-1 expression is not regulated by DAF-16. Worms carrying the *Pgsr-1::GFP* reporter construct were grown on HT115 bacteria carrying empty pL4440 vector (A, B) or subjected to *daf-16(RNAi)* (C, D). The respective effects on GFP-expression were monitored under standard culture conditions (A, C) or after induction by juglone (B, D) (quantified by ImageJ, Scale bars = 100 µm).

**Figure 6:**
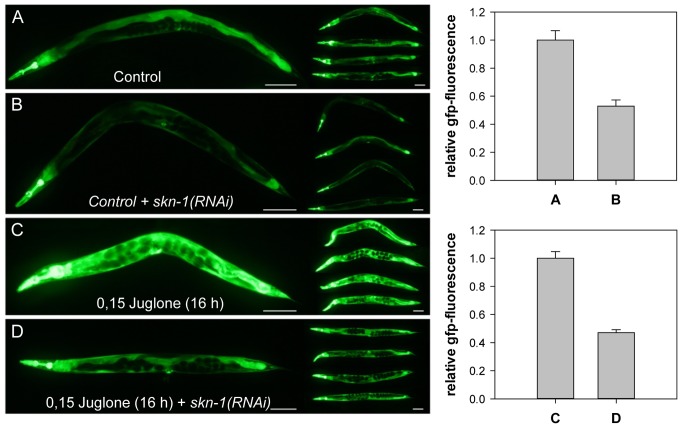
Regulation of *gsr-1* promoter activity by SKN-1. Worms carrying the *Pgsr-1::GFP* reporter construct were grown on HT115 bacteria carrying empty pL4440 vector (A, C) or subjected to *skn-1(RNAi)* (B, D). The respective effects on GFP-expression were monitored under standard culture conditions (A, B) or after induction by juglone (C, D) (quantified by ImageJ, Scale bars = 100 µm).

In *C. elegans* the transcription factors DAF-16 and SKN-1 are crucial for the coordination of stress response and both have been shown to respond to juglone [16], [28]. To analyse the contribution of these transcription factors to the regulation of the stress-responsive *gsr-1* promoter of *C. elegans*, we used the *Pgsr-1::GFP* worms. As shown in **Figure 5**, the knockdown of DAF-16 does not affect GSR-1::GFP expression under standard condition and, moreover, had no effect on the induction of GSR by juglone. Conversely, reduction of SKN-1 expression by RNAi led to a reduced GFP signal under standard conditions and to a significantly reduced induction of reporter gene expression under juglone stress (**Figure 6**). These data strongly suggest that the *gsr-1* promoter is regulated by SKN-1 and not by DAF-16. Furthermore, the obtained data indicate that SKN-1 is required for GSR-1 expression per se and for the drastic response observed under juglone stress. Accordingly, *in silico* analyses revealed three well conserved potential SKN-1 binding sites [45] at position -443, -617 and -651 of the *gsr-1* promoter region, while DAF-16 binding sites are lacking within the 1992 bp of the upstream region (**Figure S3**).

**Figure 7:**
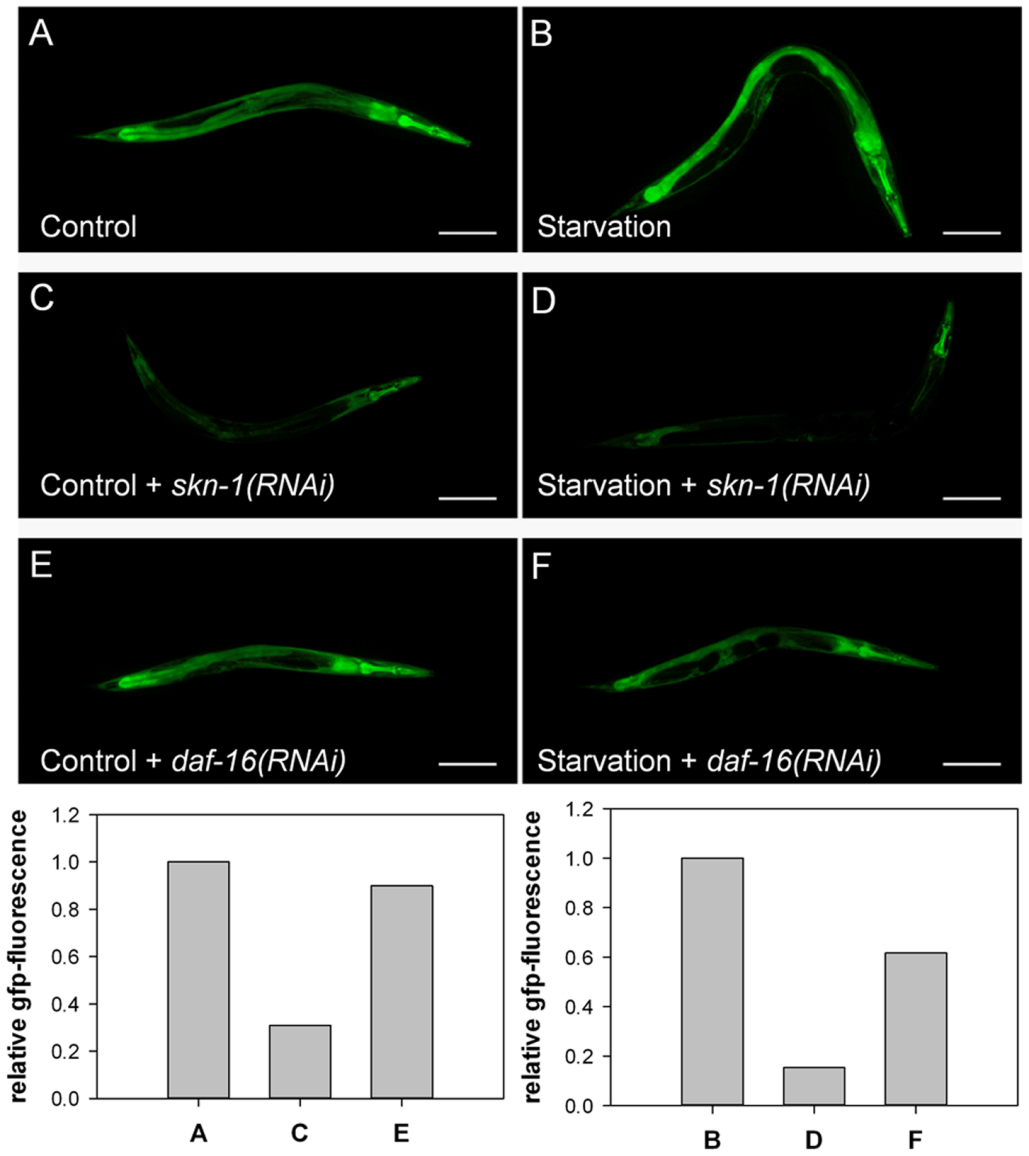
Starvation and regulation of *gsr-1* promoter activity. Worms carrying the *Pgsr-1::GFP* reporter construct were grown on HT115 bacteria carrying empty pL4440 vector (A and B) or subjected to *skn-1(RNAi)* (C and D) or *daf-16(RNAi*) (E and F). The respective effects on GFP-expression were monitored under standard culture conditions (A, C, E) or after hunger (B, D, F) (quantified by ImageJ, Scale bars = 100 µm).

In *C. elegans*, SKN-1 is also involved in the regulation of gene expression under conditions of dietary deprivation [46]. Therefore, we transferred *Pgsr-1::GFP* L4/young adult worms from standard NGM plates containing *E. coli* OP50 to starvation plates without bacterial food. As shown in **Figure 3C**, the GFP signal was considerably enhanced under starvation conditions. In good agreement with the results obtained for juglone stress, RNAi assays revealed that the observed induction of the *gsr-1* promoter activity was found to be SKN-1 but not DAF-16 dependent (**Figure 7**).

### Knockdown of gsr-1 Induces gcs-1 Expression but not Vice Versa

**Figure 8:**
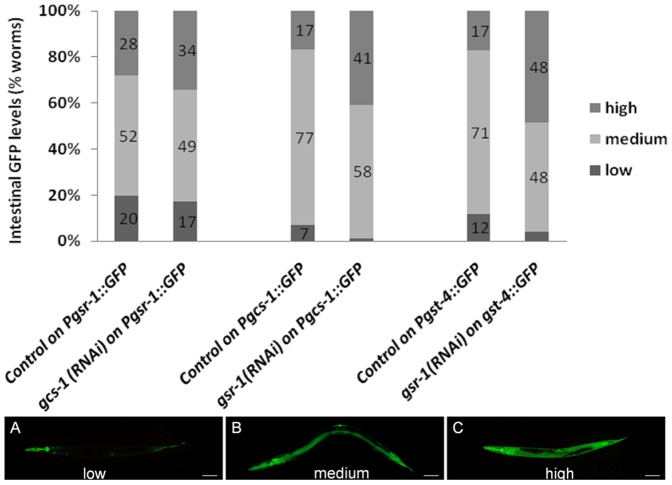
Analyses of the interrelation between GSH synthesis and GSH redox cycling. Applying RNAi, GCS-1 expression was suppressed in the *C. elegans Pgsr-1::GFP* reporter strain (left) or GSR-1 expression in the *Pgcs-1::GFP* (middle) or *Pgst-4::GFP* reporter strain (right). RNAi-treated worms were analysed by fluorescence microscopy and GFP-expression was compared with control worms grown on HT115 bacteria carrying empty pL4440 vector. Fluorescence intensities for *Pgsr-1::GFP* reporter strain were scored as low (A), medium (B) and high (C) according to [16]. Fluorescence intensity scoring for *Pgcs-1::GFP* and *Pgst-4::GFP* reporter strains is given in **Figure S4**. Significant alterations in GFP expression was detected only in *Pgcs-1::GFP* (p<0.001) and *Pgst-4::GFP* worms (p<0.001) exposed to GSR-1(RNAi). Results are the means of at least three independent assays (n = 100 worms; p values from Fisher test).

Since the cellular GSH status is regulated by redox cycling and *de novo* synthesis, we next asked whether a reduced GSH redox cycling affects GSH synthesis and, vice versa, inhibition of GSH synthesis affects GSR-1 expression. As shown in **Figure 8**, inhibition of GSH synthesis by *gcs-1(RNAi)* did not affect *gsr-1* promoter activity. In contrast to that, *gsr-1(RNAi)* resulted in a significantly enhanced GFP signal in the intestinal cells of the *Pgcs-1::GFP* reporter strain (**Figure 8** and **Figure S4 A**) indicating that GSH synthesis is induced when GSSG recycling is impaired. Under stress conditions, *gcs-1* promoter activity is stimulated by the transcription factor SKN-1 [33]. Since the expression of another SKN-1 responsive gene, *gst-4*, was similarly induced by *gsr-1(RNAi)* (**Figure 8** and **Figure S4 B**), we next analysed the nuclear translocation of a *SKN-1b/c::GFP* fusion reporter, indicative for the activation of the transcription factor. However, consistent with a recent report [47], the knockdown of *gsr-1* did not increase the nuclear accumulation of SKN-1 (data not shown). Furthermore, *gsr-1(RNAi)* did not affect the nuclear translocation of the stress responsive transcription factor DAF-16 (data not shown).

### GSR-1 Overexpression Protects against Juglone Toxicity

**Figure 9:**
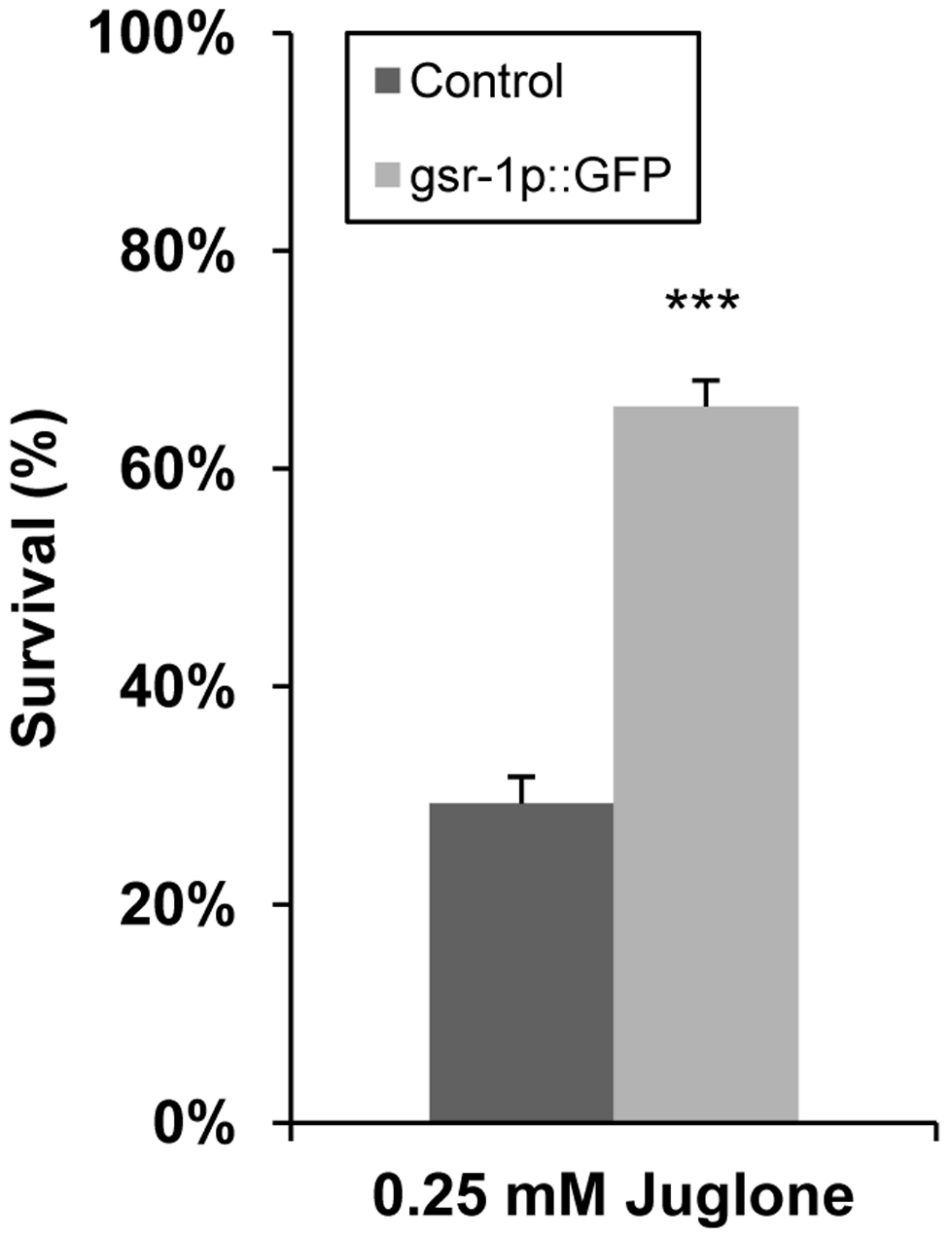
Enhanced stress tolerance by GSR-1 overexpression. L4/young adult of *C. elegans* overexpressing GSR-1::GFP fusion protein or of control worms expressing a GFP protein only were exposed to 0.25 mM juglone. Survival rate was determined after 16 h incubation. Owing to the *pha-1(e2123)* genetic background, assays were performed at 25°C. Results are means of three independent triplicate assays (n = 120 animals, Fisher test).

Since the expression level of GSR-1 was found to be elevated when worms were exposed to juglone, we next examined whether overexpression of GSR-1 is capable of increasing the stress tolerance of *C. elegans*. Transgenic *Pgsr-1::gsr-1::GFP* worms exhibited a significantly higher resistance towards juglone than the control strain (**Figure 9**). 29% of control and 66% of *gsr-1p::GFP* worms survived when exposed to 0.25 mM juglone for 16 h.

### GSR-1 Expression Level Affects Life Span

**Figure 10:**
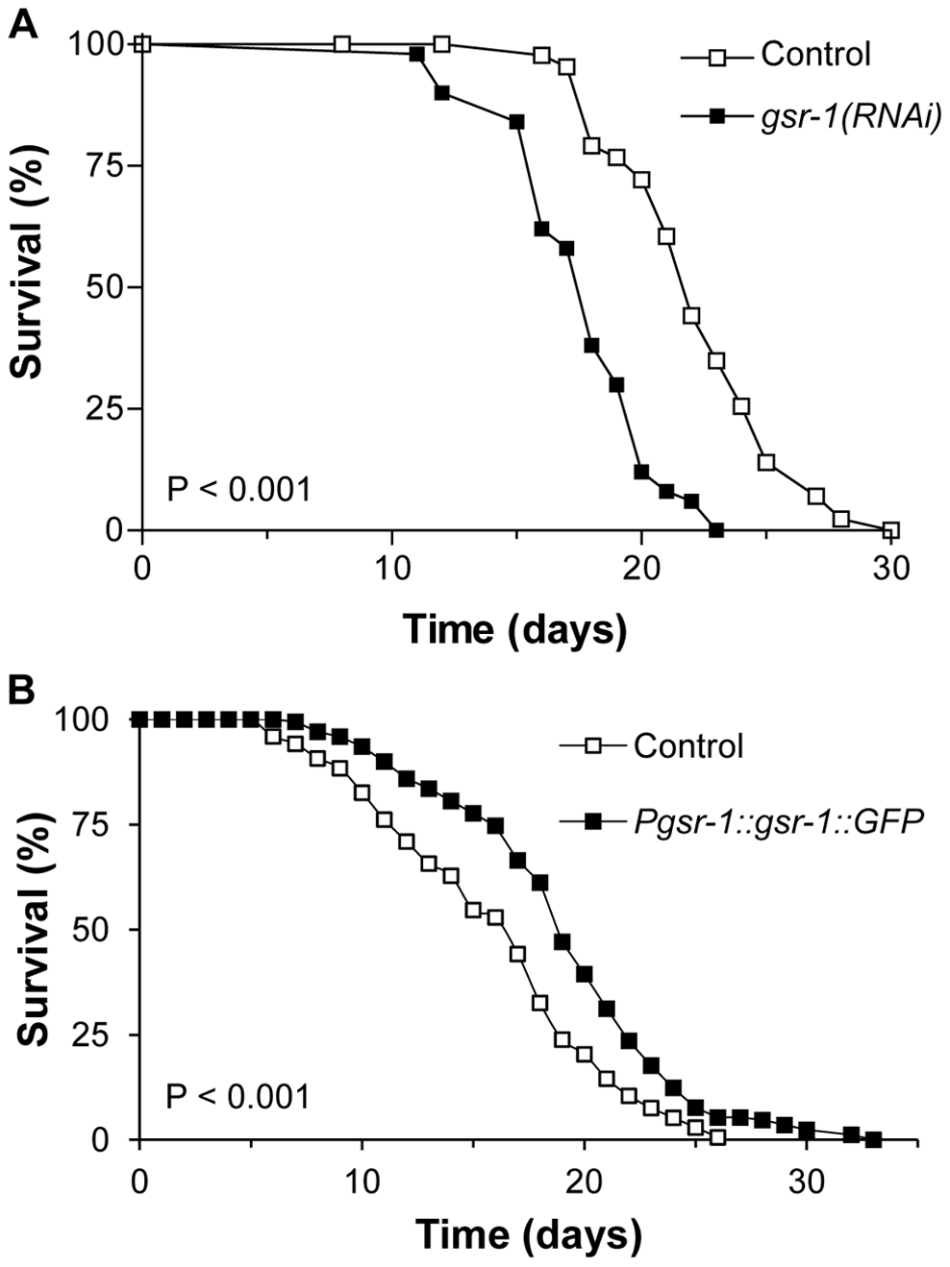
GSR-1 dependent life span. (A) *gsr-1(RNAi)* resulted in a reduced life span (log-rank test p<0.001, results are cumulative from three independent experiments with 50 worms per trial), whereas (B) overexpression of GSR-1 led to longevity (p<0.001, results are cumulative from three independent experiments with 100 worms per trial). As control, pha-1 transgenic worms were used that express GFP in a similar expression pattern (see Material and Methods).

According to the ROS theory of aging, the degree of stress resistance is often related to the life span of an organism [7], [8]. Likewise, the knockdown of GSR-1 led to a significantly reduced mean life span of 17.7 days when compared with control animals with a mean value of 20.7 days (**Figure 10A**), thus confirming the results obtained by [29].

Mean values of the 10% longest living worms were 22.4 days and 28.0 days, respectively. We observed no retardation in postembryonic development. No effects on brood size and number on progeny were observed (control: 252±35, *gsr-1(RNAi)*: 269±45; n = 30 worms).

On the other hand worms that overexpress GSR-1 displayed a prolonged life span when compared to control animals with mean values of 19.4 and 16.4 days, respectively (**Figure 10B**). Control worms had a maximal life span of 24.5 days, GSR-1 overexpressers of 27.9 days (mean values of the 10% longest living worms). The life span assay was performed at 20°C.

## Discussion

In this study we conducted a small-scale RNAi screen aiming to identify components of the GSH-metabolism that are essential for tolerance towards the pro-oxidative stressors arsenite and juglone.

In *C. elegans*, RNAi screening is a highly efficient and convenient method to determine loss-of-function phenotypes of an individual gene. A systematic analysis of the RNAi library used in this study showed that 90% of genes that were identified by classical genetics to cause embryonic lethality also showed the same phenotype using the RNAi feeding method, showing its effectiveness and quality of the library [48]. However, it is important to note that the effectiveness of RNAi in the suppression of gene expression can be variable and we cannot confirm a complete abrogation of gene function for each RNAi clone used that did not cause a detectable phenotype. Additionally, our RNAi experiments cannot exclude combined effects of several enzymes. However, we can be sure of the RNAi clones that showed a detectable phenotype, like the *gsr-1*, *gst-44*, *gst-32*, *gst0-1*, *gcs-1* and the extracellular GPX. Interestingly, a recent publication clearly demonstrates the combined action of thioredoxin reductase and GSR-1 during molting. Here the regulated reduction of cuticle components is driven by thioredoxin reductase and GSR-1 [49].

Arsenic-mediated toxicity is thought to be due to oxidative stress on the one hand and on the other hand due to biotransformation by methylation and accumulation of the metalloid in the cells. As described before, tolerance towards arsenite is primarily GSH synthesis-dependent, with p38 MAPK being activated by arsenite treatment, the PMK-1 phosphorylating the transcription factor SKN-1, thereby inducing its nuclear translocation and the activation of *gcs-1* gene expression [32], [43], [44]. Additionally, our RNAi screen indicated the involvement of an antioxidant protein, the extracellular GPX (C11E4.1) and two phase II xenobiotic-metabolizing enzymes, the *gst-32* and *gst-44*. Both, the alpha-class related *gst-32* and the GPX are implicated in the oxidative stress response by way of their GSH-dependent reduction of inorganic and organic peroxides or by their ability to eliminate secondary products of lipid peroxidation. However, the role of *gst-44* remains unclear. Since methylation of arsenic involves an omega-class GST that functions as a monomethylarsonate reductase [22], an involvement of *gst-44* in arsenic biotransformation seems feasible. Also, the enzyme is exclusively localizes in the cytoplasm of the H-shaped cell associated with the excretory system. Besides osmoregulation and secretion of molting fluid or homones, one of the proposed functions of the excretory system is the excretion of metabolic waste (Wormatlas database) However, like *Drosophila melanogaster*, *C. elegans* does not possess an arsenic methyltransferase homolog, making methylation processes of inorganic arsenic highly unlikely to occur in *C. elegans* [50]. Another possible function of the omega-class GST could be the reduction of GSH-mixed disulphides once oxidative stress has been removed. Knockdown of the GSR-1 does not seem to affect arsenite sensitivity. However, as mentioned above, negative RNAi results must be treated with caution since small amounts of residual GSR-1 might be sufficient for arsenite tolerance. Literature search also gave ambiguous results. For example, exposure of porcine endothelial cells to trivalent arsenics caused an increase in total glutathione levels and higher enzymatic activity of GPX and GST, with no increase being observed in GSR-activity [51]. Whereas in rice roots, the enzymatic activity of the GSR responded in a dose-dependent manner to arsenic [52], in ferns the GSR acitivity remained unchanged, with the GR not being inhibited or activated by arsenite in enzymatic assays [53].

The toxicity of juglone is due to redox cycling as well as direct electrophilic interaction with glutathione [22]. In our small-scale RNAi-screen, only the GSR-1 was identified to be absolutely essential for survival under sub-lethal juglone stress. This indicates that the maintenance of low GSSG concentrations is vital to the preservation of an adequate reduction potential when exposed to increasing oxidative stress.

At our applied juglone concentration, no changes in the total GSH level of stressed control worms were found, however, enhanced GSSG- and γ-GC levels and a reduced GSH/GSSG ratio were observed. Under oxidative insult, the existing GSH pool is oxidized to GSSG by nonenzymatic or enzymatic routes. Here GPX uses GSH to reduce peroxides, while GRX reduces protein disulphides directly via its active site dithiol, which is converted to a disulphide needing to be reduced again by GSH and thereby producing GSSG. The availability of GSH is ensured by GSSG recyling and/or by de novo synthesis, both of which are up-regulated when oxidative stress occurs [54]. Another protective mechanism during oxidative insult is the rapid and active transportation of excessive GSSG out of the cell, demonstrating the acute need of the cell to prevent its toxic accumulation [55]. Using our current experimental setup of derivatising whole worms, we cannot detect whether active GSSG export is taking place during juglone stress and/or paired with *gsr-1(RNAi),* but this can be the explanation of why we observe constant total GSH levels. However, the observed changes in γ-GC levels strongly indicate that de novo GSH synthesis is boosted under juglone stress and that it is potentiated by simultaneous *gsr-1(RNAi)*.

During juglone stress, *gsr-1(RNAi)* aggravates intracellular oxidative challenge, as any GSSG generated in the cell cannot be reduced back to GSH, thereby severely limiting the efficient removal of peroxides by GPX or the reduction of protein disulphides by GRX, which is at the expense of GSH to GSSG. Therefore, these functions can only be performed until the supply of GSH is exhausted. This explains the enormous effect of *gsr-1* knockdown on the tolerance towards juglone stress observed in the above mentioned results. Likewise, overexpression of GSR-1 is capable of increasing the stress tolerance of *C. elegans* towards juglone. Arsenite also reinforces the generation of oxidative stress, but this arsenite-generated oxidative stress and the one generated by juglone seems to have critical different properties. Wherease juglone induces the expression of GSR-1 capable of reducing GSSG, arsenite does not. In conclusion, GSH synthesis and redox cycling are crucial for different stressors and cannot compensate each other.

Overexpression of the GSR-1 is associated with an enhanced resistance to pro-oxidant stress and the boosting of the capacity to maintain critical GSH/GSSG ratios may underlie this beneficial effect on survival. Furthermore, overexpression of GSR-1 is associated with an extension of life span, perhaps due to improvement of the in vivo response to age-related oxidative stress. GSH/GSSG fluctuations, that might occur due to increasing ROS levels accompanying aging, might be deleterious at the level of antioxidant defenses and/or redox-sensitive signal transduction. During the aging process in mice, the GSH/GSSG ratio becomes progressively more pro-oxidizing due to an increase in the GSSG content and a decline in the *de novo* GSH synthesis. Experimental studies by Rebrina & Sohal [56] suggest that the age-related accumulation of homocystein is causing a loss of affinity between the GCS and its substrates. In *Drosophila*, the GSSG content and amounts of protein mixed disulphides increases during aging, while GSH concentrations remained unaffected. Interestingly, neuronal overexpression of GCS extends life span up to 50% [57]. Although highly controversial [58], the free radical theory of aging predicts that resistance to oxidative stress is often associated with life span extension and our findings reinforce this concept. On the other hand, according to [59], oxidative stress may have dose-dependent opposing effects on life span: a mild increase of mitochondrial superoxide levels, e.g. in *C. elegans* sod-2 deletion mutants, was shown to have prosurvival effects, whereas higher superoxide levels were associated with toxic effects. Furthermore, it was shown that under mild stress conditions increasing antioxidative capacity could have negative effects on survival. However, redox state may be more important for longevity than stress tolerance *per se.*

In evolutionary terms, target genes that are at the endpoints of a stress response cascade are not the ones whose actions significantly influence the operations of other genes. However, factors that act at early stages of the cascade are critical for influencing other cell functions. Therefore, it was surprising that the knockdown of GSR-1 had an influence on GCS-1 expression. An impaired GSSG recycling caused by gsr-1(RNAi) affects the GSH redox state and in response to changes in the GSH/GSSG ratio, reversible S-glutathionylation of proteins can occur, thereby possibly preventing the irreversible oxidation of protein thiols or the loss of glutathione by the rapid GSSG extrusion from the cell. S-glutathionylation, however, can also result in protein-specific functional changes that regulate specific cellular processes or act as a redox switch in signalling pathways [60]. Hence, it is intriguing to speculate that a disturbance of the GSH/GSSG ratio - in our case by *gsr-1(RNAi)* - affects the SKN-1 dependent expression of GCS-1 via such an S-glutathionylation mechanism in order to replenish the GSH pool and maintain a high GSH/GSSG ratio. A probable mediator is the WD40 repeat protein WDR-23. WDR-23 contains 17 Cys-residues and has been previously considered as a redox sensor regulating SKN-1 function in concert with the ubiquitin ligase complex CUL4/DDB1 [61]. Hence, the intracellular redox state is suggested to be maintained by a SKN-1/WDR-23-dependent autoregulatory loop.

## Supporting Information

Figure S1 **Recombinant expression and biochemical characterisation of** ***C. elegans*** **GSR-1.**
(DOCX)

Figure S2 ***Gsr-1(RNAi)*** **on** ***gsr-1p::GFP*** **worms.**
(DOCX)

Figure S3 **GSR-1 promoter region.** Putative SNK-1 conserved binding sites were manually determined using the consensus sequence for SKN-1 binding WWTRTCAT.
(DOCX)

Figure S4 **Representative fluorescence intensities for** ***Pgcs-1::GFP*** **reporter strain and** ***Pgst-4::GFP*** **reporter strain are shown.**
(DOCX)

## References

[1] Sies H (1993) Strategies of antioxidant defense. Eur J Biochem 215: 213–219. 7688300 10.1111/j.1432-1033.1993.tb18025.x

[2] Davies KJ (2000) Oxidative stress, antioxidant defenses, and damage removal, repair, and replacement systems. IUBMB Life 50: 279–289. 11327322 10.1080/713803728

[3] Valko M, Leibfritz D, Moncol J, Cronin MT, Mazur M, et al (2007) Free radicals and antioxidants in normal physiological functions and human disease. Int J Biochem Cell Biol 39: 44–84. 16978905 10.1016/j.biocel.2006.07.001

[4] Limon-Pacheco J, Gonsebatt ME (2009) The role of antioxidants and antioxidant-related enzymes in protective responses to environmentally induced oxidative stress. Mutat Res 674: 137–147. 18955158 10.1016/j.mrgentox.2008.09.015

[5] Harman D (1956) Aging: a theory based on free radical and radiation chemistry. J Gerontol 11: 298–300. 13332224 10.1093/geronj/11.3.298

[6] Sohal RS, Weindruch R (1996) Oxidative stress, caloric restriction, and aging. Science 273: 59–63. 8658196 10.1126/science.273.5271.59 PMC2987625

[7] Hekimi S, Guarente L (2003) Genetics and the specificity of the aging process. Science 299: 1351–1354. 12610295 10.1126/science.1082358

[8] Muller FL, Lustgarten MS, Jang Y, Richardson A, Van Remmen H (2007) Trends in oxidative aging theories. Free Radic Biol Med 43: 477–503. 17640558 10.1016/j.freeradbiomed.2007.03.034

[9] Gems D, Doonan R (2009) Antioxidant defense and aging in *C. elegans*: is the oxidative damage theory of aging wrong? Cell Cycle 8: 1681–1687. 19411855 10.4161/cc.8.11.8595

[10] Perez VI, Bokov A, Van Remmen H, Mele J, Ran Q, et al (2009) Is the oxidative stress theory of aging dead? Biochim Biophys Acta 1790: 1005–1014. 19524016 10.1016/j.bbagen.2009.06.003 PMC2789432

[11] Townsend DM, Tew KD, Tapiero H (2003) The importance of glutathione in human disease. Biomed Pharmacother 57: 145–155. 12818476 10.1016/s0753-3322(03)00043-x PMC6522248

[12] Zhang H, Forman HJ, Choi J (2005) Gamma-glutamyl transpeptidase in glutathione biosynthesis. Methods Enzymol 401: 468–483. 16399403 10.1016/S0076-6879(05)01028-1

[13] Ishii N (2005) The role of stress in ageing: research on the nematode, *Caenorhabditis elegans* . Br J Dermatol 153 Suppl 21–5. 10.1111/j.1365-2133.2005.06963.x 16280015

[14] Baumeister R, Schaffitzel E, Hertweck M (2006) Endocrine signaling in *Caenorhabditis elegans* controls stress response and longevity. J Endocrinol 190: 191–202. 16899554 10.1677/joe.1.06856

[15] Braeckman BP, Vanfleteren JR (2007) Genetic control of longevity in *C. elegans.* . Exp Gerontol 42: 90–98. 16829009 10.1016/j.exger.2006.04.010

[16] Tullet JM, Hertweck M, An JH, Baker J, Hwang JY, et al (2008) Direct inhibition of the longevity-promoting factor SKN-1 by insulin-like signaling in *C. elegans* . Cell 132: 1025–1038. 18358814 10.1016/j.cell.2008.01.030 PMC2367249

[17] Lant B, Storey KB (2010) An overview of stress response and hypometabolic strategies in *Caenorhabditis elegans*: conserved and contrasting signals with the mammalian system. Int J Biol Sci 6: 9–50. 20087441 10.7150/ijbs.6.9 PMC2808051

[18] Jomova K, Jenisova Z, Feszterova M, Baros S, Liska J, et al (2011) Arsenic: toxicity, oxidative stress and human disease. J Appl Toxicol 31: 95–107. 21321970 10.1002/jat.1649

[19] Lee TC, Wei ML, Chang WJ, Ho IC, Lo JF, et al (1989) Elevation of glutathione levels and glutathione S-transferase activity in arsenic-resistant Chinese hamster ovary cells. In Vitro Cell Dev Biol 25: 442–448. 2732199 10.1007/BF02624629

[20] Kumagai Y, Sumi D (2007) Arsenic: signal transduction, transcription factor, and biotransformation involved in cellular response and toxicity. Annu Rev Pharmacol Toxicol 47: 243–262. 17002598 10.1146/annurev.pharmtox.47.120505.105144

[21] Thomas DJ (2009) Unraveling arsenic–glutathione connections. Toxicol Sci 107: 309–311. 19074764 10.1093/toxsci/kfn257

[22] Inbaraj JJ, Chignell CF (2004) Cytotoxic action of juglone and plumbagin: a mechanistic study using HaCaT keratinocytes. Chem Res Toxicol 17: 55–62. 14727919 10.1021/tx034132s

[23] Strayer A, Wu Z, Christen Y, Link CD, Luo Y (2003) Expression of the small heat-shock protein Hsp16–2 in *Caenorhabditis elegans* is suppressed by *Ginkgo biloba* extract EGb 761. FASEB J 17: 2305–2307. 14525938 10.1096/fj.03-0376fje

[24] Wang X, Thomas B, Sachdeva R, Arterburn L, Frye L, et al (2006) Mechanism of arylating quinone toxicity involving Michael adduct formation and induction of endoplasmic reticulum stress. Proc Natl Acad Sci U S A 103: 3604–3609. 16505371 10.1073/pnas.0510962103 PMC1450130

[25] Burmeister C, Luersen K, Heinick A, Hussein A, Domagalski M, et al (2008) Oxidative stress in *Caenorhabditis elegans*: protective effects of the Omega class glutathione transferase (GSTO-1). FASEB J 22: 343–354. 17901115 10.1096/fj.06-7426com

[26] Kahn NW, Rea SL, Moyle S, Kell A, Johnson TE (2008) Proteasomal dysfunction activates the transcription factor SKN-1 and produces a selective oxidative-stress response in *Caenorhabditis elegans* . Biochem J 409: 205–213. 17714076 10.1042/BJ20070521

[27] Hartwig K, Heidler T, Moch J, Daniel H, Wenzel U (2009) Feeding a ROS-generator to *Caenorhabditis elegans* leads to increased expression of small heat shock protein HSP-16.2 and hormesis. Genes Nutr 4: 59–67. 19252938 10.1007/s12263-009-0113-x PMC2654055

[28] Przybysz AJ, Choe KP, Roberts LJ, Strange K (2009) Increased age reduces DAF-16 and SKN-1 signaling and the hormetic response of *Caenorhabditis elegans* to the xenobiotic juglone. Mech Ageing Dev 130: 357–369. 19428455 10.1016/j.mad.2009.02.004 PMC2680786

[29] Spanier B, Rubio-Aliaga I, Hu H, Daniel H (2010) Altered signalling from germline to intestine pushes daf-2;pept-1 *Caenorhabditis elegans* into extreme longevity. Aging Cell 9: 636–646. 20550516 10.1111/j.1474-9726.2010.00591.x

[30] Brenner S (1974) The genetics of *Caenorhabditis elegans* . Genetics 77: 71–94. 4366476 10.1093/genetics/77.1.71 PMC1213120

[31] Lewis JA, Fleming JT, McLafferty S, Murphy H, Wu C (1987) The levamisole receptor, a cholinergic receptor of the nematode *Caenorhabditis elegans* . Mol Pharmacol 31: 185–193. 3807894

[32] An JH, Blackwell TK (2003) SKN-1 links *C. elegans* mesendodermal specification to a conserved oxidative stress response. Genes Dev 17: 1882–1893. 12869585 10.1101/gad.1107803 PMC196237

[33] An JH, Vranas K, Lucke M, Inoue H, Hisamoto N, et al (2005) Regulation of the *Caenorhabditis elegans* oxidative stress defense protein SKN-1 by glycogen synthase kinase-3. Proc Natl Acad Sci U S A 102: 16275–16280. 16251270 10.1073/pnas.0508105102 PMC1283458

[34] Ahinger J (2006) Reverse genetics *WormBook*, ed. The C. elegans Research community, doi/10.1895/wormbook.1.47.1, http://www.wormbook.org.

[35] Granato M, Schnabel H, Schnabel R (1994) pha-1, a selectable marker for gene transfer in *C. elegans* . Nucleic Acids Res 22: 1762–1763. 8202383 10.1093/nar/22.9.1762 PMC308061

[36] Clos J, Brandau S (1994) pJC20 and pJC40–two high-copy-number vectors for T7 RNA polymerase-dependent expression of recombinant genes in *Escherichia coli.* . Protein Expr Purif 5: 133–137. 8054844 10.1006/prep.1994.1020

[37] Bradford MM (1976) A rapid and sensitive method for the quantitation of microgram quantities of protein utilizing the principle of protein-dye binding. Anal Biochem 72: 248–254. 942051 10.1016/0003-2697(76)90527-3

[38] Muller S, Gilberger TW, Fairlamb AH, Walter RD (1997) Molecular characterization and expression of *Onchocerca volvulus* glutathione reductase. Biochem J 325 (Pt 3): 645–651. 10.1042/bj3250645 PMC1218607 9271084

[39] Neuschwander-Tetri BA, Roll FJ (1989) Glutathione measurement by high-performance liquid chromatography separation and fluorometric detection of the glutathione-orthophthalaldehyde adduct. Anal Biochem 179: 236–241. 2774172 10.1016/0003-2697(89)90121-8

[40] Oliveira RP, Porter Abate J, Dilks K, Landis J, Ashraf J, et al (2009) Condition-adapted stress and longevity gene regulation by *Caenorhabditis elegans* SKN-1/Nrf. Aging Cell 8: 524–541. 19575768 10.1111/j.1474-9726.2009.00501.x PMC2776707

[41] Uberbacher EC, Mural RJ (1991) Locating protein-coding regions in human DNA sequences by a multiple sensor-neural network approach. Proc Natl Acad Sci U S A 88: 11261–11265. 1763041 10.1073/pnas.88.24.11261 PMC53114

[42] Claros MG, Vincens P (1996) Computational method to predict mitochondrially imported proteins and their targeting sequences. Eur J Biochem 241: 779–786. 8944766 10.1111/j.1432-1033.1996.00779.x

[43] Inoue H, Hisamoto N, An JH, Oliveira RP, Nishida E, et al (2005) The *C. elegans* p38 MAPK pathway regulates nuclear localization of the transcription factor SKN-1 in oxidative stress response. Genes Dev 19: 2278–2283. 16166371 10.1101/gad.1324805 PMC1240035

[44] Liao VH, Yu CW (2005) *Caenorhabditis elegans* gcs-1 confers resistance to arsenic-induced oxidative stress. Biometals 18: 519–528. 16333752 10.1007/s10534-005-2996-3

[45] Blackwell TK, Bowerman B, Priess JR, Weintraub H (1994) Formation of a monomeric DNA binding domain by Skn-1 bZIP and homeodomain elements. Science 266: 621–628. 7939715 10.1126/science.7939715

[46] Paek J, Lo JY, Narasimhan SD, Nguyen TN, Glover-Cutter K, et al (2012) Mitochondrial SKN-1/Nrf mediates a conserved starvation response. Cell Metab 16: 526–537. 23040073 10.1016/j.cmet.2012.09.007 PMC3774140

[47] Wang J, Robida-Stubbs S, Tullet JM, Rual JF, Vidal M, et al.. (2010) RNAi screening implicates a SKN-1-dependent transcriptional response in stress resistance and longevity deriving from translation inhibition. PLoS Genet 6. 10.1371/journal.pgen.1001048 PMC2916858 20700440

[48] Ahringer J (1996) Posterior patterning by the *Caenorhabditis elegans* even-skipped homolog vab-7. Genes Dev 10: 1120–1130. 8654927 10.1101/gad.10.9.1120

[49] Stenvall J, Fierro-Gonzalez JC, Swoboda P, Saamarthy K, Cheng Q, et al (2011) Selenoprotein TRXR-1 and GSR-1 are essential for removal of old cuticle during molting in *Caenorhabditis elegans* . Proc Natl Acad Sci U S A 108: 1064–1069. 21199936 10.1073/pnas.1006328108 PMC3024696

[50] Rizki M, Kossatz E, Velazquez A, Creus A, Farina M, et al (2006) Metabolism of arsenic in *Drosophila melanogaster* and the genotoxicity of dimethylarsinic acid in the *Drosophila* wing spot test. Environ Mol Mutagen 47: 162–168. 16304668 10.1002/em.20178

[51] Yeh JY, Cheng LC, Ou BR, Whanger DP, Chang LW (2002) Differential influences of various arsenic compounds on glutathione redox status and antioxidative enzymes in porcine endothelial cells. Cell Mol Life Sci 59: 1972–1982. 12530527 10.1007/PL00012519 PMC11337438

[52] Ahsan N, Lee DG, Alam I, Kim PJ, Lee JJ, et al (2008) Comparative proteomic study of arsenic-induced differentially expressed proteins in rice roots reveals glutathione plays a central role during As stress. Proteomics 8: 3561–3576. 18752204 10.1002/pmic.200701189

[53] Kertulis-Tartar GM, Rathinasabapathi B, Ma LQ (2009) Characterization of glutathione reductase and catalase in the fronds of two *Pteris* ferns upon arsenic exposure. Plant Physiol Biochem 47: 960–965. 19574057 10.1016/j.plaphy.2009.05.009

[54] Circu ML, Aw TY (2008) Glutathione and apoptosis. Free Radic Res 42: 689–706. 18671159 10.1080/10715760802317663 PMC3171829

[55] Park HA, Khanna S, Rink C, Gnyawali S, Roy S, et al (2009) Glutathione disulfide induces neural cell death via a 12-lipoxygenase pathway. Cell Death Differ 16: 1167–1179. 19373248 10.1038/cdd.2009.37 PMC2990696

[56] Rebrin I, Sohal RS (2008) Pro-oxidant shift in glutathione redox state during aging. Adv Drug Deliv Rev 60: 1545–1552. 18652861 10.1016/j.addr.2008.06.001 PMC2585506

[57] Orr WC, Radyuk SN, Prabhudesai L, Toroser D, Benes JJ, et al (2005) Overexpression of glutamate-cysteine ligase extends life span in *Drosophila melanogaster* . J Biol Chem 280: 37331–37338. 16148000 10.1074/jbc.M508272200

[58] Speakman JR, Selman C (2011) The free-radical damage theory: Accumulating evidence against a simple link of oxidative stress to ageing and lifespan. Bioessays 33: 255–259. 21290398 10.1002/bies.201000132

[59] Van Raamsdonk JM, Hekimi S (2012) Superoxide dismutase is dispensable for normal animal lifespan. Proc Natl Acad Sci U S A 109: 5785–5790. 22451939 10.1073/pnas.1116158109 PMC3326508

[60] Dalle-Donne I, Rossi R, Colombo G, Giustarini D, Milzani A (2009) Protein S-glutathionylation: a regulatory device from bacteria to humans. Trends Biochem Sci 34: 85–96. 19135374 10.1016/j.tibs.2008.11.002

[61] Choe KP, Przybysz AJ, Strange K (2009) The WD40 repeat protein WDR-23 functions with the CUL4/DDB1 ubiquitin ligase to regulate nuclear abundance and activity of SKN-1 in *Caenorhabditis elegans* . Mol Cell Biol 29: 2704–2715. 19273594 10.1128/MCB.01811-08 PMC2682033

