# Supplementary material for: The Glutathione Reductase GSR-1 Determines Stress Tolerance and Longevity in Caenorhabditis elegans
Source: PLoS One. 2013 Apr 8;8(4):e60731. doi: 10.1371/journal.pone.0060731 (PMC3620388; doi:10.1371/journal.pone.0060731)

**Figure S1.** Recombinant expression and biochemical characterisation of *C. elegans* GSR-1. (A) Recombinant *C. elegans* GSR-1 containing an N-terminal His_6_-tag was purified by Ni-NTA agarose (lane 1) followed by gel filtration on a Sephadex S100 column (lane 3). Lane 2, protein standards. (B) Michaelis-Menten and Lineweaver-Burk plots of recombinant *C. elegans* GSR-1 for NADPH and GSSG.


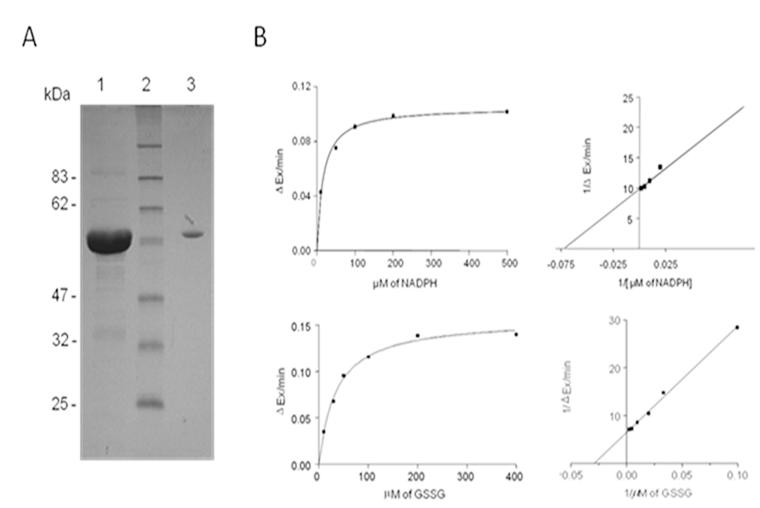

Supplement: Figure S1 — Recombinant expression and biochemical characterisation of C. elegans GSR-1. (DOCX) [file pone.0060731.s001.docx]
