# Supplementary material for: The Glutathione Reductase GSR-1 Determines Stress Tolerance and Longevity in Caenorhabditis elegans
Source: PLoS One. 2013 Apr 8;8(4):e60731. doi: 10.1371/journal.pone.0060731 (PMC3620388; doi:10.1371/journal.pone.0060731)
Supplement: Figure S2 — Gsr-1(RNAi) on gsr-1p::GFP worms. (DOCX) [file pone.0060731.s002.docx]

**Figure S2.** *Gsr-1(RNAi)* on *gsr-1p::GFP* worms. (A) Fluorescent micrographs of untreated *gsr-1p::GFP* worms, (B) treatment of *gsr-1p::GFP* worms with *gsr-1(RNAi)* effectively reduces GFP expression (C) Western blot analysis. To comfirm equal loading, one part of the SDS-gel was stained with Coomassie brilliant blue stain (top panel), the other part was blotted and probed with anti-GFP (bottom panel). Control worms were grown in the presence of an empty pL4440 vector (lane 1). *Pgsr-1::gsr-1::GFP* worms without the application of *gsr-1(RNAi)* (lane 2) and following *gsr-1(RNAi)*. Clearly a reduction of GSR::GFP fusion protein is observed (lane 3).

**C**

**
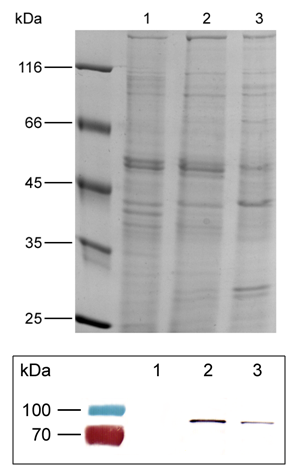
**
