# Supplementary material for: The Glutathione Reductase GSR-1 Determines Stress Tolerance and Longevity in Caenorhabditis elegans
Source: PLoS One. 2013 Apr 8;8(4):e60731. doi: 10.1371/journal.pone.0060731 (PMC3620388; doi:10.1371/journal.pone.0060731)
Supplement: Figure S3 — GSR-1 promoter region. Putative SNK-1 conserved binding sites were manually determined using the consensus sequence for SKN-1 binding WWTRTCAT. (DOCX) [file pone.0060731.s003.docx]

**Figure S3.** GSR-1 promoter region cloned for the two reporter gene constructs *Pgsr-1::GFP* and *Pgsr-1::gsr-1::GFP*. Putative SNK-1 conserved binding sites were manually determined using the consensus sequence for SKN-1 binding WWTRTCAT (32). The first eight nucleotides of the coding region are in capitals, the three putative SKN-1 binding sites are underlined and in bold.

ccatcatttcgggactcgacattccggtgtcgtagagagattttagaagaaatctggaaaaatttaataggattaagccg -1913

tatgtgtcgatttacgcaagtcgtgtactcctcgagaagaagaagaacacagttttcggctaaaattcttgatttggaac -1833

tgtttgagctaattttttctgtattcgagtttagagcaccgttttgtagttacagtagttttcgtgacgggacccaaatt -1753

ggtttagcacccgtcgagctgtattcaaaattcctgctgaaaatagaaaaaggattttttaatcatgttttctatacttt -1673

ttttgttttttgaagttttttacagcaaaagaattggcgaagatggttcaaaatagatcaaaatatacccaaaagttgtg -1593

aatttttaaaaataaaatgcaaacttctcttcgaggagtacactttttatgttgggaattttcctcacgtcagaacattt -1513

ttcttatcaccaggtgtgcattggatattcttatcagccaatactaatttatcatgaacactttgagatggctgagcgac -1433

ggttggacggagacagaatgtcgttcggaaatttatcattggacggtagaactctaatgcactgaaaaaattattatttt -1353

catttcattattttcaattttccagccgtactctgcataaatccattttctcagattggcggcgttcaccgctgatacga -1273

aataatctcttttactcttccagatttgtttacgccgagccaatggatactcttcatctattccaagttccaagtcgagt -1193

tgatcccaagcacggcgagacaaattcgaattccgatgacaaatcaggcgttgatcccataggactaaaaagggaaattt -1113

ttggaaaaacttcttgatttcctgacttactaggccttttccgaactgcatatataatatcgtacactttttcaggctcc -1033

gcgtaatcgatttttgtagattggcggacccgattgtacttaaaatacttgtctgagcgtgtttgcacagtcaaatcact -953

gaaaaaaggataaaaaagaaatctgataataaatgttttaattcatactttccatcattcttgagcatatcggctgtaat -873

tgaaggaatgtacatcgaattgttggatgctcgataataaactgtttcgggaataattccggcatcagttttcgctttta -793

attcttcgtaatttgtctgaaatctttgaatagaattgaaacgaatattataaaaagctgtaccggaatattgcgacaag -713

atcttctagttggtccaagacgaagtttcttcgattcgctggcgctctgaaaaaatgtttg**atttgtcat**cattttttct -633

agttttcagtgaaac**ttttgtcat**catttattagttttttcttgtgcctgattgaattgattgaaactacatataaataa -553

tcactattttaattttattgcccactcgcgctctttttggtttttttctaataaaattgtctttcttctctgcgcactcc -473

actattgaccaaatcagtgtttctttct**atttgtcat**tgccccgacactgcgaagaacgttcgcgattgttctttgtctt -393

ttttacctctcttaattcctccgttgtaattgatttcaagtcgtcatcgagttgttgaaaatcggcaaacacttttcgct -313

tcactattcggccgcttcgagatttttcaacttctgaaaaattgagagattcatatagaccatttttcaacatttacctg -233

tcattttttattccagcgtgcgaatccgtttggaaaattgagcggaatttttaatttccaaaaaaacttctaaatttcaa -153

attttaaactcttcgcgcgctgaaaaaggggcggagttttcaattcgcaaaatgggcgcgagctatttgcaacttgggcg -73

cacaatttagtccttgatgtttgtttattttttgctgatcaaatgtctaaaaacttctgtgaacccttctga**ATGCTCCG** +8
