# Supplementary material for: The Glutathione Reductase GSR-1 Determines Stress Tolerance and Longevity in Caenorhabditis elegans
Source: PLoS One. 2013 Apr 8;8(4):e60731. doi: 10.1371/journal.pone.0060731 (PMC3620388; doi:10.1371/journal.pone.0060731)
Supplement: Figure S4 — Representative fluorescence intensities for Pgcs-1::GFP reporter strain and Pgst-4::GFP reporter strain are shown. (DOCX) [file pone.0060731.s004.docx]

**Figure S4.** Fluorescence intensities for *Pgcs-1::GFP* reporter strain (upper) and *Pgst-4::GFP* reporter strain (lower). Reporter strains were scored as low (A), medium (B) and high (C) according to Tullet *et al*. (2008).


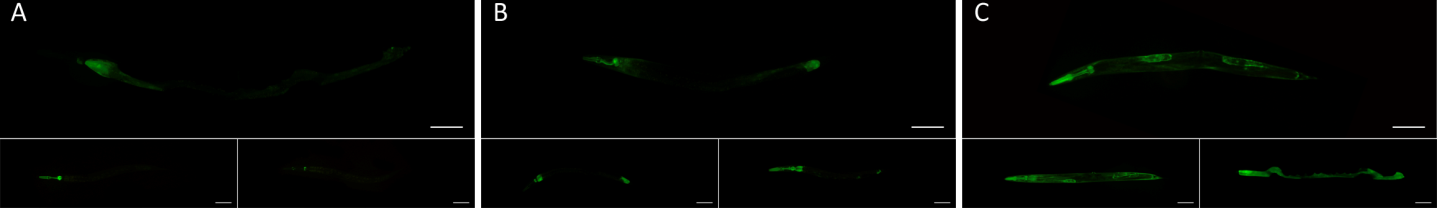


**
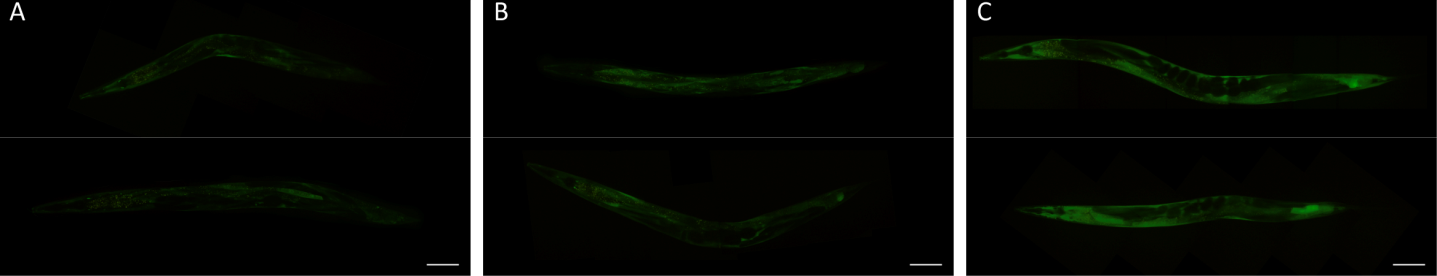
**
